# Activity-regulated trafficking of the palmitoyl-acyl transferase DHHC5

**DOI:** 10.1038/ncomms9200

**Published:** 2015-09-03

**Authors:** G. Stefano Brigidi, Brendan Santyr, Jordan Shimell, Blair Jovellar, Shernaz X. Bamji

**Affiliations:** 1 Department of Cellular and Physiological Sciences, and the Djavad Mowafaghian Center for Brain Health, University of British Columbia, 2350 Health Sciences Mall, Vancouver, British Columbia, Canada, V6T-1Z3

## Abstract

Synaptic plasticity is mediated by the dynamic localization of proteins to and from synapses. This is controlled, in part, through activity-induced palmitoylation of synaptic proteins. Here we report that the ability of the palmitoyl-acyl transferase, DHHC5, to palmitoylate substrates in an activity-dependent manner is dependent on changes in its subcellular localization. Under basal conditions, DHHC5 is bound to PSD-95 and Fyn kinase, and is stabilized at the synaptic membrane through Fyn-mediated phosphorylation of a tyrosine residue within the endocytic motif of DHHC5. In contrast, DHHC5's substrate, δ-catenin, is highly localized to dendritic shafts, resulting in the segregation of the enzyme/substrate pair. Neuronal activity disrupts DHHC5/PSD-95/Fyn kinase complexes, enhancing DHHC5 endocytosis, its translocation to dendritic shafts and its association with δ-catenin. Following DHHC5-mediated palmitoylation of δ-catenin, DHHC5 and δ-catenin are trafficked together back into spines where δ-catenin increases cadherin stabilization and recruitment of AMPA receptors to the synaptic membrane.

## Abstract (web summary)

Synaptic plasticity is mediated by the dynamic localization of proteins at synapses, which is partly controlled via palmitoylation of synaptic proteins. Here, the authors show how neuronal activity regulates the palmitoylation reaction through the translocation of the palmitoyl-acyl transferase DHHC5.

## Graphical abstract

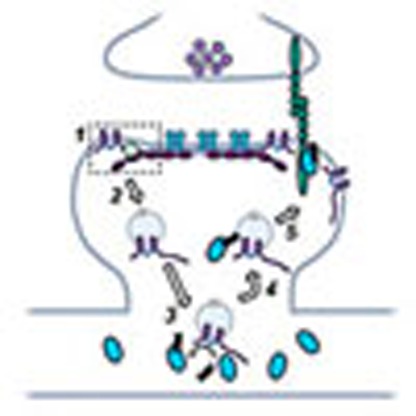

Synapses of the central nervous system are highly plastic structures that are modified in response to fluctuations in neuronal activity. Changes in the number, size and composition of synapses have been observed following alterations in neuronal activity *in vitro*^1^,^2^,^3^ and following the learning of specific tasks *in vivo*^4^,^5^. Thus, elucidating the molecular mechanisms underlying activity-mediated trafficking of proteins to and from synaptic compartments is essential for our understanding of brain function.

Palmitoylation is a reversible posttranslational modification involving the addition of palmitate onto cysteine residues that facilitates the trafficking of proteins to cell membranes^6^. Protein palmitoylation is mediated by a family of multi-pass, transmembrane palmitoyl-acyl transferase (PAT) enzymes that contain a zinc-finger domain and a conserved Asp-His-His-Cys (DHHC, also called zDHHC) motif that is required for palmitoyl-transferase activity^7^. In neurons, DHHC proteins are localized to the Golgi, vesicular or plasma membranes, and palmitate cycling on substrate proteins can be constitutive or dynamically regulated by cell signals^8^,^9^. Recent work has demonstrated that a number of synaptic proteins are substrates for palmitoylation, and that activity regulates the palmitoylation and trafficking of these proteins^7^,^10^.

DHHC5 is localized to postsynaptic compartments^11^ and can palmitoylate Grip1b^11^, δ-catenin^12^, Flotillin-2 (ref. 13), somatostatin receptor 5 (ref. 14) and Ankyrin-G^15^. DHHC5-mediated palmitoylation of Grip1b and δ-catenin increases synaptic delivery and surface stabilization of α-amino-3-hydroxy-5-methyl-4-isoxazole propionic acid receptors (AMPARs), respectively, implicating DHHC5 in the regulation of synapse efficacy^11^,^12^.

The importance of DHHC5 in synaptic regulation is supported by impaired synapse plasticity and performance on learning and memory tasks in mice homozygous for a hypomorphic allele of the *ZDHHC5* gene^16^. Genome-wide association studies have reported the occurrence of mutations within a region of chromosome 11 containing *ZDHHC5* in patients with bipolar disorders and schizophrenia^17^,^18^. Moreover, a *de novo* nonsense mutation in the DHHC5 protein has also recently been reported in schizophrenic patients^19^, indicating a possible involvement of DHHC5 in these neuropsychiatric disorders.

We have previously shown that activity increases DHHC5-mediated palmitoylation of δ-catenin^12^. Here we demonstrate that this is not due to alterations in the enzymatic activity of DHHC5 but rather its subcellular localization. Under basal conditions, DHHC5 is stabilized at the synaptic membrane through its association with PSD-95 and Fyn kinase. This occurs through Fyn-mediated phosphorylation of DHHC5 at tyrosine 533 and the inhibition of DHHC5 endocytosis. DHHC5 is subsequently stabilized at synapses and sequestered from its substrate, δ-catenin, which is primarily localized to dendritic shafts. Neuronal activity disrupts the DHHC5/PSD-95/Fyn kinase complex and enhances the internalization and trafficking of DHHC5 from spines to dendritic shafts where it binds and palmitoylates δ-catenin. We demonstrate that DHHC5 is mobilized on recycling endosomes (REs) and is subsequently re-trafficked back into spine synapses together with δ-catenin. Our findings demonstrate that activity-dependent regulation of DHHC protein trafficking provides a mechanism for the local control of protein palmitoylation and delivery to synapses.

## Results

### Neuronal activity does not alter DHHC5 autopalmitoylation

**Figure 1:**
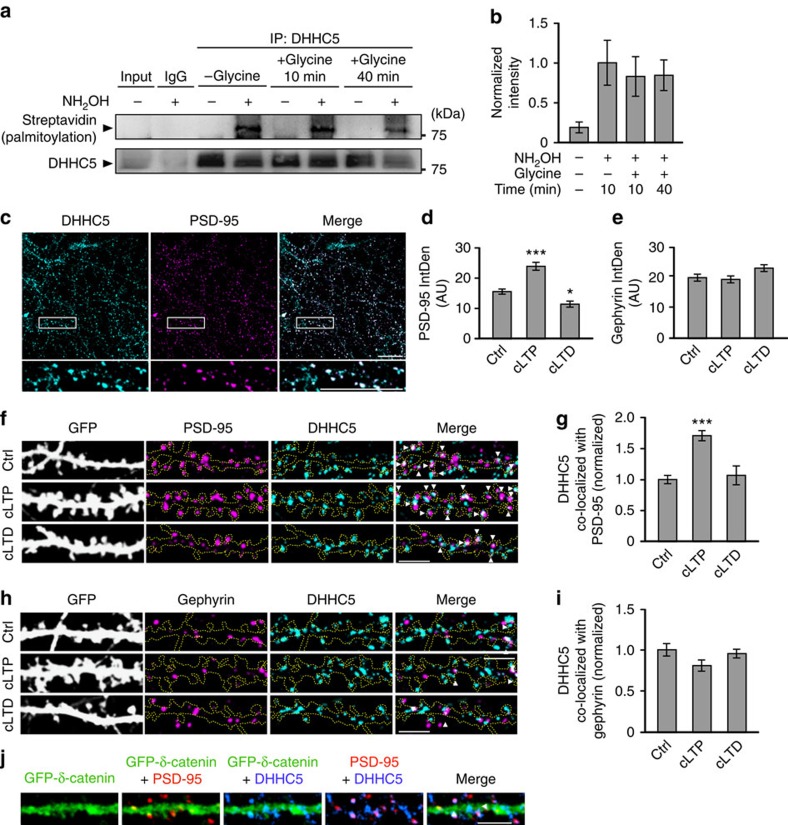
Changes in DHHC5 palmitoylation and localization following increased neuronal activity. (**a**,**b**) Autopalmitoylation of DHHC5 is not altered following cLTP (glycine) stimulation (*P*=0.107, F_3,8_=2.83, *n*=3 blots from 3 cultures). Exclusion of NH_2_OH was used as a control for the specificity of biotin labelling. (**c**) Confocal image of 14 DIV neurons demonstrating co-localization of DHHC5 and PSD-95. (**d**,**e**) The IntDen of PSD-95 puncta is altered 40 min after treatment with cLTP and cLTD relative to control cells (ctrl), (*P*<0.001, F_2,84_=15.28, *n*=38, 35, 14), whereas the IntDen of gephyrin is not (*P*=0.354, F_2,93_=1.05, *n*=37, 39, 20). Confocal images of 14 DIV neurons (**f**,**h**) demonstrating increased co-localization of DHHC5 with PSD-95 (*P*<0.001, F_2,84_=22.98) (**f**,**g**), but no change in co-localization of DHHC5 and gephyrin (*P*=0.114, F_2,93_=2.21) (**h**,**i**) 40 min after cLTP. Co-localized puncta are denoted by white arrowheads. (**j**) Confocal images of 14 DIV neurons transfected with GFP–δ-catenin and immunostained for PSD-95 and DHHC5 (*n*=45 cells). Scale bars, 20 μm (**c**) and 5 μm (**f**,**h**,**j**). *n*=cells from three separate cultures. All graphs display mean±s.e.m. *P<0.05, ***P<0.001; one-way analysis of variance; Tukey's *post-hoc* test. (**a**) Five per cent of whole-cell lysates was loaded as input. Full-length blots of **a** presented in Supplementary Fig. 5.

We have previously shown that neuronal activity enhances DHHC5-mediated palmitoylation of its substrate, δ-catenin^12^. To further understand the molecular mechanism underlying this process, we first determined whether activity enhances protein palmitoylation by increasing the enzymatic activity of DHHC5. Recent analysis of DHHC PATs indicates that protein *S*-palmitoylation proceeds by a two-step mechanism: the initial autopalmitoylation of a DHHC cysteine side chain followed by the transfer of palmitate to the substrate cysteine^7^,^20^. Therefore, the autopalmitoylation of DHHC proteins can be used as a surrogate measure of enzymatic activity^8^,^20^. We examined whether neuronal activity enhances the palmitoylation of DHHC5 using the acyl-biotin exchange (ABE) assay, which exchanges palmitoyl modifications with biotin and can therefore determine bulk palmitoylation levels^10^,^21^ (Fig. 1a). Exclusion of hydroxylamine (NH_2_OH) was used as a control for the specificity of biotin labelling, as it is essential for the cleavage of palmitate from cysteines^11^,^12^,^22^. Fourteen days *in vitro* (DIV) hippocampal neurons were stimulated using a standard chemical long-term potentiation (cLTP) protocol involving a 3-min treatment with glycine/bicucculine that selectively activates synaptic *N*-methyl-D-aspartate receptors (NMDARs). This has been shown to recruit AMPARs to the synaptic membrane and enhance synapse strength in both dissociated hippocampal cultures^2^,^12^,^23^,^24^ and slices^25^. There was no significant difference in the palmitoylation of DHHC5 10 and 40 min after stimulation with glycine, time points associated with activity-induced palmitoylation of δ-catenin by DHHC5 (ref. 12 and Fig. 1a,b), suggesting that activity does not regulate the enzymatic function of DHHC5.

### Neuronal activity regulates DHHC5 subcellular localization

We next examined whether neuronal activity controls the palmitoylation of substrates by modifying the subcellular localization of DHHC enzymes. Under basal conditions, 56.9±5.8% of endogenous DHHC5 co-localized with the postsynaptic protein PSD-95 (Fig. 1c,f, *n*=14 cells), a faithful marker of excitatory synapses (Supplementary Fig. 1a,b). DHHC5 (31.18±2.53%) co-localized with the inhibitory postsynaptic protein gephyrin (Fig. 1h, *n*=32 cells), a faithful marker of inhibitory synapses (Supplementary Fig. 1c,d). Together, this indicates that ∼88% of DHHC5 is localized to synapses. Furthermore, 79.73±4.92% of excitatory synapses and 46.78±1.77% of inhibitory synapses co-localize with DHHC5, indicating that the majority of synapses contain DHHC5.

We stimulated 14 DIV neurons using cLTP^2^ or chemical long-term depression (cLTD)^26^ protocols in which cells were treated for 3 min with glycine/bicucculine or glycine/NMDA, respectively, and then returned to basal media for 40 min. In contrast to cLTP, cLTD activates both synaptic and extrasynaptic NMDARs, resulting in AMPAR internalization and synaptic depression^26^,^27^. The efficacy of these protocols in our cells were confirmed by determining the integrated density (IntDen; product of area and mean grey value) and density of PSD-95 puncta, which was significantly increased following cLTP and decreased following cLTD relative to unstimulated control cells (ctrl), in agreement with previous observations (Fig. 1d and Supplementary Fig. 1e)^2^,^26^,^28^. No changes were observed in the IntDen or density of gephyrin following cLTP or cLTD (Fig. 1e and Supplementary Fig. 1f). Interestingly, the IntDen of DHHC5 within spines was significantly increased 40 min following cLTP but not cLTD (in AU: –Gly, 14.51±0.46, *n*=38 cells; +Gly, 19.02±0.86, *n*=35; +Gly/NMDA, 15.18±1.07, *n*=14; *P*<0.001, F_2,84_=12.99, one-way analysis of variance).

DHHC5/PSD-95 co-localization also increased 40 min after cLTP (Fig. 1f,g), whereas DHHC5/gephyrin co-localization was unaffected (Fig. 1h,i). Interestingly, cLTD did not affect DHHC5 co-localization with either synaptic marker (Fig. 1f–i). Together, this indicates that cLTP increases the recruitment of DHHC5 specifically to excitatory spine synapses, whereas cLTD does not affect DHHC5 localization at either excitatory or inhibitory synapses.

### Activity-induced trafficking of DHHC5

We next focused on how activity-mediated translocation of DHHC5 impacts its ability to palmitoylate its synaptic substrates. As Grip1b palmitoylation is not activity regulated^8^,^11^, we focused on δ-catenin, which is palmitoylated by DHHC5 following activity^12^. Under basal conditions, 55.79±2.48% of DHHC5 and 28.57±1.31% of green fluorescent protein (GFP)–δ-catenin are localized to spine synapses (GFP–δ-catenin was validated in ref. 12). Only 27.31±0.05% of GFP–δ-catenin co-localized with DHHC5, with virtually all co-localization occurring at spine synapses (93.38±6.34% DHHC5/δ-catenin co-localized with PSD-95; Fig. 1j, *n*=45 cells). This demonstrates that DHHC5 and δ-catenin are largely localized to separate compartments with a fraction of DHHC5 and δ-catenin localized to spines, in accordance with the low level of δ-catenin palmitoylation and spine localization under basal conditions^12^.

**Figure 2:**
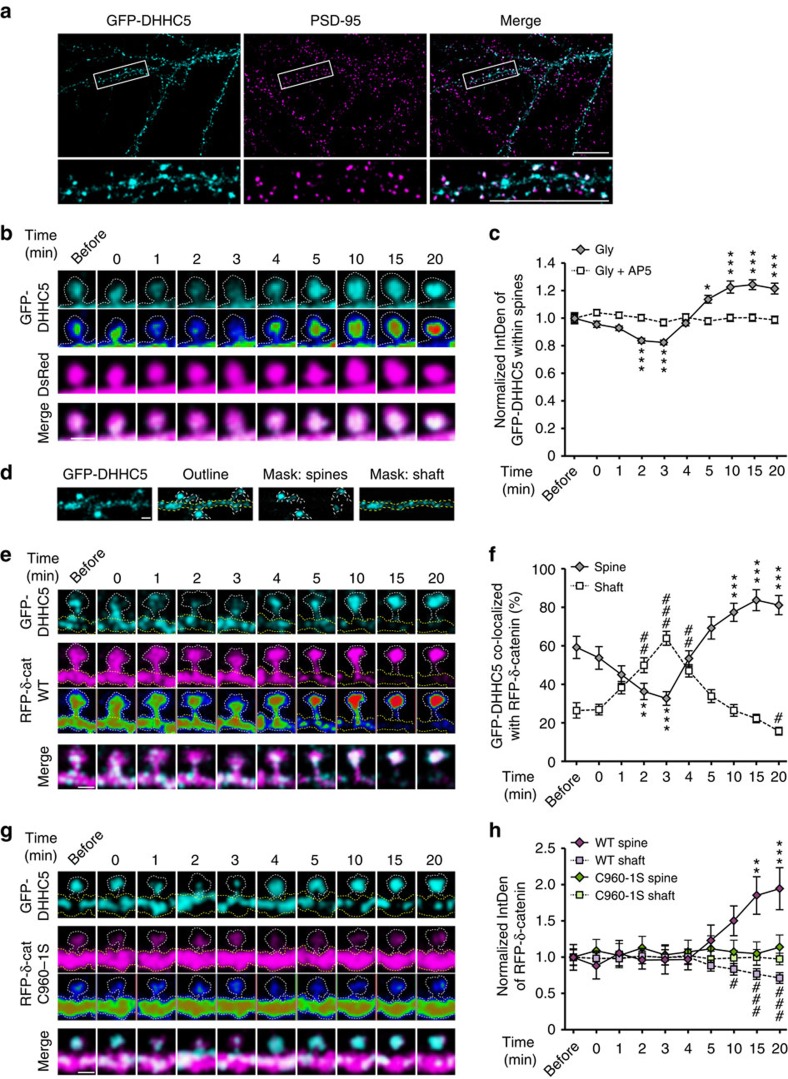
Activity enhances DHHC5 trafficking from spines. (**a**) Confocal images of 14 DIV neurons demonstrating partial co-localization of GFP–DHHC5 and PSD-95. (**b**) High-magnification confocal images of GFP–DHHC5 fluorescence (lower panels pseudocolored as a heat map) and DsRed before and after glycine stimulation. (**c**) GFP–DHHC5 fluorescence decreases transiently within spines after glycine stimulation (*P*<0.001, F_9,220_=42.45; *n*=221 spines, 8 cells). Treatment with AP5, DL-2-Amino-5-phosphonopentanoic acid, abolishes this (*P*=0.46, F_9,141_=0.974, *n*=142 spines, 6 cells). (**d**) Representative image of GFP–DHHC5 within masks made of spines (dashed white line) or dendritic shaft (dashed yellow line). High-magnification confocal images of GFP–DHHC5 and RFP–δ-catenin WT (**e**) or C960-1S (**g**; lower panels pseudocoloured as a heat map) within a single spine and region of dendrite shaft (traced with white and yellow dashed lines, respectively) before and after stimulation. (**f**) GFP–DHHC5 co-localized with RFP–δ-catenin WT decreases in spines (*P*<0.001, F_9,6_=91.14, *n*=7 cells) and increases in shafts (*P*<0.001, F_9,6_=58.24, *n*=7 cells) transiently following cLTP. (**h**) RFP–δ-catenin WT is recruited to spines (*P*<0.001, F_9,6_=7.549, *n*=7 cells) and is depleted from shafts (*P*<0.001, F_9,6_=11.83, *n*=7 cells) following activity, whereas RFP–δ-catenin C960-1S is unchanged in both spines (*P*=0.43, F_9,6_=1.025, *n*=7 cells) and shafts (*P*=0.64, F_9,6_=0.783, *n*=7 cells). *n*=number of cells or spines from three to five separate cultures. Scale bars, 20 μm (**a**) and 1 μm (**b**,**d**,**e**,**g**). All graphs show mean±s.e.m. (**c**,**f**,**h**). Asterisks and cross-hatches (**f**,**h**) above data points indicate significance relative to before stimulation within spines or dendrites, respectively. *P<0.05, **/^##^*P*<0.01, ***/^###^*P*<0.001; repeated-measures one-way analysis of variance, Tukey's *post-hoc* test.

To track the localization of both δ-catenin and DHHC5 following activity, we transfected cells with GFP–DHHC5 and red fluorescent protein (RFP)–δ-catenin. We first confirmed that GFP–DHHC5 is a faithful marker of DHHC5 by comparing its localization at synapses with that of endogenous DHHC5 (Fig. 2a). GFP–DHHC5 (49.4±5.0%) localized to excitatory synapses (*n*=19 cells), which was statistically similar to the 55.79±2.48% of endogenous DHHC5 localized to synapses (Fig. 1c; *P*=0.343, Student's *t*-test). GFP–DHHC5 puncta were localized to spine heads before cLTP treatment (now indicated in the figures as the absence or the presence of glycine treatment; ±Gly), translocated out of spines 2–3 min after stimulation and then trafficked back into spines 4 min after stimulation with significantly more DHHC5 localized to spines 5–20 min after stimulation (Fig. 2b,c). Stimulation of cells in the presence of the NMDAR blocker, DL-2-amino-5-phosphonopentanoic acid (AP5) (50 μM), abolished glycine-mediated translocation of GFP–DHHC5 (Fig. 2c). Masks of spines and dendritic shafts were made to quantify all data (Fig. 2d).

As previously shown, expressing GFP–DHHC5 in neurons increased the localization of δ-catenin to spines even under basal conditions resulting in the localization of RFP–δ-catenin in both spines and shafts (Fig. 2e)^12^. Two to three minutes after stimulation, GFP–DHHC5 was significantly less co-localized with wild-type (WT) RFP–δ-catenin within spines and the two were significantly more co-localized in dendritic shafts (Fig. 2e,f). GFP–DHHC5 translocated back into spines together with RFP–δ-catenin WT 3–20 min after stimulation (Fig. 2e,f), resulting in the accumulation of WT RFP–δ-catenin in spines and the depletion of RFP–δ-catenin WT from shafts (Fig. 2e,h). In contrast, the palmitoylation-deficient RFP–δ-catenin C960-1S mutant^12^ was virtually absent from spines, even in cells expressing GFP–DHHC5, and did not translocate to spines post stimulation (Fig. 2g,h). Moreover, the trafficking of GFP–DHHC5 was unaffected in cells expressing the δ-catenin C960-1S mutant (Fig. 2g), indicating that DHHC5 trafficking is independent of δ-catenin. Together, this demonstrates that DHHC5 is driven out of spines following cLTP and then trafficked back into spines together with palmitoylated δ-catenin.

### Activity-induced endocytosis and trafficking of DHHC5

**Figure 3:**
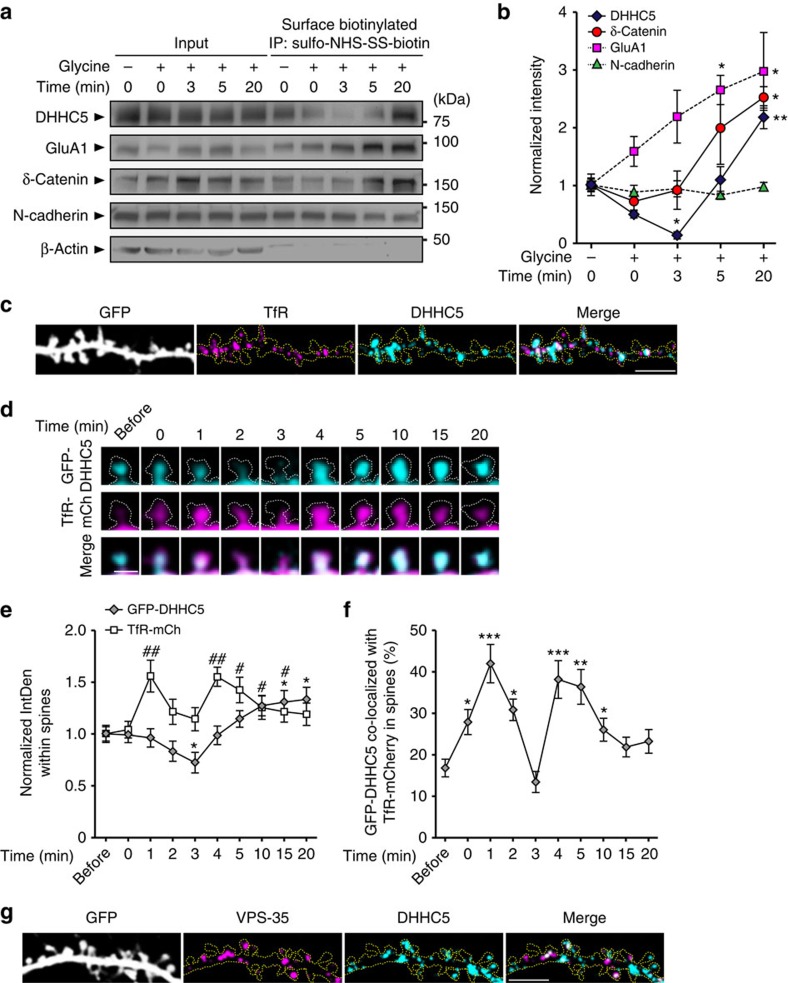
Activity-induced DHHC5 endocytosis and trafficking on recycling endosomes. (**a**,**b**) Fourteen DIV hippocampal neurons were stimulated with cLTP and then biotinylated at the indicated time points. Lysates were immunoprecipitated with neutravidin-coated beads to isolate all surface proteins and blots probed with the indicated antibodies. (**b**) Intensity of protein levels in the surface fraction, normalized to whole-cell input levels. DHHC5: *P*<0.001, F_4,10_=24.35; δ-catenin: *P*=0.016, F_4,10_=5.16; GluA1: *P*=0.036, F_4,10_=3.933; N-cadherin: *P*=0.724, F_4,10_=0.52). *n*=3 blots, 3 cultures. (**c**) Confocal image of 14 DIV hippocampal neurons demonstrating co-localization between TfR and DHHC5 (*n*=38 cells). (**d**) High-magnification confocal images of GFP–DHHC5 and TfR-mCh fluorescence in a single spine (white dashed line) over time (*n*=6 cells, 3 cultures). (**e**) IntDen of GFP–DHHC5 (*P*<0.001, F_9,45_=7.21) and TfR-mCh (*P*=0.002, F_9,45_=4.71) within spines. (**f**) Per cent GFP–DHHC5 co-localized with TfR-mCh in spines (*P*<0.001, F_9,45_=7.21). (**g**) Confocal image of hippocampal neurons demonstrating co-localization between VPS-35 and DHHC5 at 14 DIV (*n*=22 cells). Scale bars, 5 μm (**c**,**g**) and 1 μm (**d**). Graphs show mean±s.e.m. (**b**) *P<0.05, **P<0.01; one-way analysis of variance (ANOVA), Tukey's *post-hoc* test, (**e**,**f**) */#*P*<0.05, **/^##^*P*<0.01, ***P<0.001; repeated-measures one-way ANOVA, Tukey's *post-hoc* test. Asterisks and cross-hatches (**e**) above data points indicate significance relative to before stimulation for GFP–DHHC5 and TfR-mCherry, respectively. (**a**) Fifty per cent of whole-cell lysates were loaded as inputs. Full-length blots are presented in Supplementary Fig. 5.

DHHC proteins have previously been shown to localize to both the cell and RE membranes^9^,^11^,^29^,^30^. Using a biotinylation assay, we demonstrated that DHHC5 is localized to the cell surface under basal conditions (Fig. 3a,b). cLTP stimulation decreased surface DHHC5 levels 3 min post stimulation, followed by the return of DHHC5 to the membrane 5–20 min after stimulation. DHHC5 surface levels were significantly enhanced compared with baseline 20 min after cLTP (Fig. 3a,b), in accordance with the increased amount of DHHC5 observed in spine synapses 20 min after cLTP (Fig. 2f,g). To confirm the specificity of DHHC5 biotinylation, we mutated the extracellular arginine residue to alanine (R182) and demonstrated a lack of biotinylation, despite its plasma membrane localization (Supplementary Fig. 2a–c). We also demonstrated that DHHC5 is directly biotinylated and not pulled down in a complex with other biotinylated proteins (Supplementary Fig. 2d).

As expected, cLTP enhanced surface GluA1 levels^22^,^31^, whereas N-cadherin levels remained unchanged^12^ (Fig. 3a,b). We also observed an accumulation of δ-catenin in the surface fraction 3–20 min post stimulation (Fig. 3a,b). As δ-catenin is a purely cytosolic protein, this result suggests that δ-catenin is recruited to the membrane where it binds to and co-immunoprecipitates with biotinylated membrane proteins such as N-cadherin. This interpretation was further confirmed by repeating the assay in increasing salt concentrations (Supplementary Fig. 2e–h). As an imporant negative control, very little cytosolic β-actin was detected in the surface fraction (Fig. 3a and Supplementary Fig. 2e,i).

The internalization and recycling of surface proteins is a highly regulated process that can be modified by synaptic activity through regulation of dendritic endosomal pathways^32^,^33^,^34^,^35^. To determine whether DHHC5 is trafficked on REs, we determined DHHC5 localization with respect to the transferrin receptor (TfR)^1^,^28^,^33^. We demonstrated that 39.74±2.07% of DHHC5 co-localized with TfR (*n*=38 cells), indicating that a fraction of endogenous DHHC5 is localized to dendritic REs under basal conditions (Fig. 3c). Immediately following stimulation (0–1 min), TfR-mCherry (mCh) rapidly translocated into spines (Fig. 3d,e) and was significantly more co-localized with GFP–DHHC5 (Fig. 3d,f). Two to three minutes after stimulation, both GFP–DHHC5 and TfR-mCh trafficked out of spines as observed by both a decrease in their IntDen (Fig. 3d,e) and their decreased co-localization within spines (Fig. 3d,f). The two proteins trafficked together back into spines 3–4 min after stimulation as evidenced by their increased IntDen (Fig. 3d,e) and their increased co-localization within spines (Fig. 3d,f). GFP–DHHC5 continued to accumulate in spines 5–20 min post stimulation, whereas TfR-mCh was trafficked out. TfR-positive REs have previously been shown to rapidly traffic in and out of spines following enhanced activity^1^,^28^. Our results also demonstrate rapid translocation of TfR-positive REs and further demonstrate that endocytosed DHHC5 is transported to dendritic shafts on REs.

To confirm the transport of DHHC5 between plasma and RE membranes, we immunostained for VPS-35, a marker for the retromer complex that mediates trafficking of protein cargos between these two compartments^36^. DHHC5 (49.04±3.76%) was localized to VPS-35 puncta in dendrites (spines plus shafts) (Fig. 3g), providing further support that DHHC5 traffics between the plasma membrane and REs.

### DHHC5 binds δ-catenin and regulates its synaptic delivery

**Figure 4:**
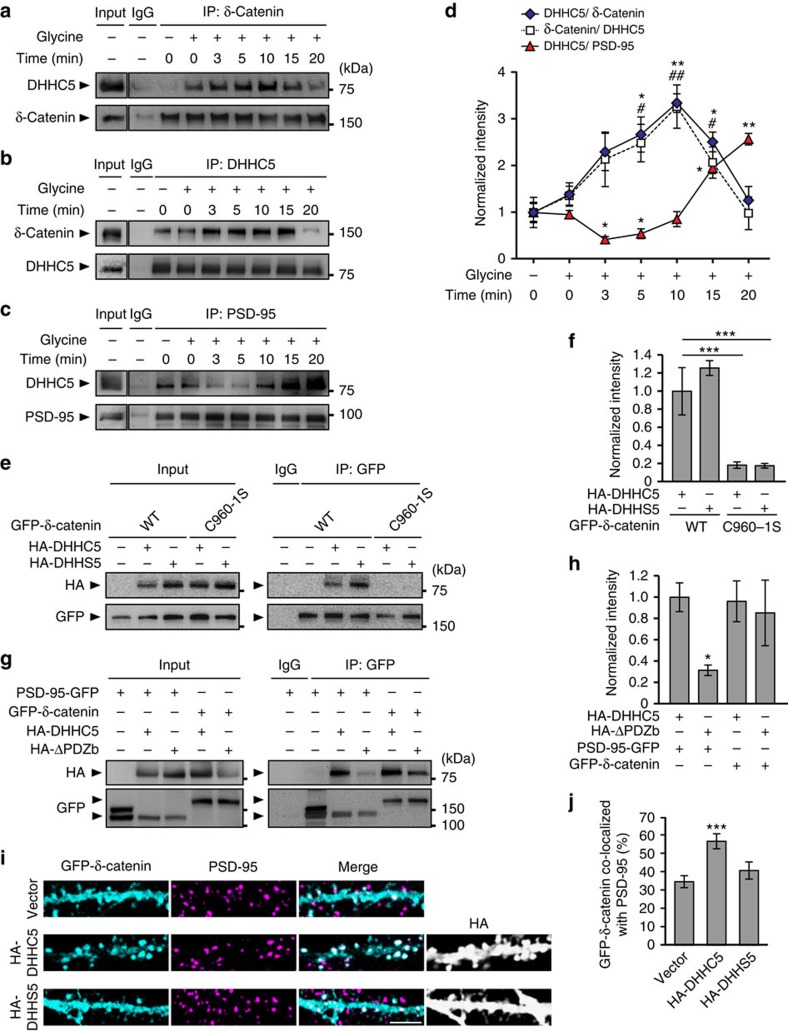
Activity regulates the association of DHHC5 with δ-catenin and PSD-95. (**a**–**d**) Fourteen to 16 DIV hippocampal neurons were stimulated, lysed at the indicated time points, lysates immunoprecipitated and blots probed with the indicated antibodies. (**a**,**d**) DHHC5–δ-catenin interactions increase transiently following cLTP (*P*=0.0006, F_6,14_=8.172). (**b**,**d**) Reverse co-IPs show the same (*P*=0.004, F_6,14_=4.98). (**c**,**d**) PSD-95 /DHHC5 interactions decrease transiently following cLTP (*P*<0.001, F_6,14_=32.89). *n*=3 blots from 3 separate cultures. (**e**–**h**) HEK293T cells were transfected with HA-DHHC5, GFP–δ-catenin or PSD-95-GFP constructs, for 36 h. Lysates were immunoprecipitated using anti-GFP and blots probed with the indicated antibodies. (**e**,**f**) DHHC5 WT associated with δ-catenin WT or C960-1S (*P*=0.0011, F_3,8_=7.46). (**g**,**h**) DHHC5 WT and ΔPDZb associated with PSD-95 or δ-catenin (*P*=0.019, F_3,8_=6.65). *n*=3 blots from 3 separate cultures. (**i**) Confocal image of 14 DIV neurons transfected with GFP–δ-catenin and HA-DHHC5 or DHHS5, and immunostained for PSD-95. Scale bar, 5 μm. (**j**) Per cent GFP–δ-catenin co-localized with PSD-95 (*P*<0.001, F_2,59_=8.54; *n*=24 (vector), 20 (HA-DHHC5) and 18 (HA-DHHS5) cells, 3 cultures). All graphs display mean±s.e.m. */^#^*P*<0.05, **/^##^*P*<0.01, ***P<0.001; one-way analysis of variance; Tukey's *post-hoc* test. Asterisks and cross-hatches (**d**) above data points indicate significance relative to unstimulated controls for DHHC5/δ-catenin or DHHC5/PSD-95 and δ-catenin/DHHC5, respectively. (**a**,**b**,**c**,**e**,**g**) Five percent of whole-cell lysate were loaded as inputs, and (**a**,**b**,**c**) are from the same blots but with different exposure times. Full-length blots are presented in Supplementary Fig. 5.

DHHC proteins have been shown to bind to their substrates during palmitoylation^11^; hence, we determined whether the time course for DHHC5/δ-catenin interactions corresponded with the translocation of δ-catenin into spine synapses. We hypothesized that DHHC5 may bind to substrates, palmitoylate them and orchestrate their translocation from one subcellular compartment to the next. Under basal conditions, DHHC5–δ-catenin interactions were low, in agreement with the low levels of palmitoylated δ-catenin observed in the absence of activity^12^. DHHC5–δ-catenin interactions were significantly increased 5–10 min after cLTP and then returned to basal levels by 15 min (Fig. 4a,b,d). The activity-induced increase in DHHC5–δ-catenin interactions (5–15 min, Fig. 4d) corresponded with a decrease in DHHC5–PSD-95 interactions (3–5 min, Fig. 4c,d) and coincided with the endocytosis (3 min, Fig. 3a,b) and trafficking of DHHC5 out of spines (2–5 min, Figs 2b–g,3a,b,d–f).

To determine how DHHC5 regulates the trafficking of δ-catenin into spines, we examined how these two proteins interact. We demonstrated that the palmitoylation-defective C960-1S δ-catenin mutant does not bind to DHHC5 (Fig. 4e,f), suggesting that δ-catenin binds to DHHC5 through an interface involving its palmitoylated cysteine residues. We also demonstrated that the ability of DHHC5 to bind to δ-catenin does not depend on either its autopalmitoylation (as DHHS5 can bind to δ-catenin WT) (Fig. 4e,f) or its PDZ-binding domain (as DHHC5 ΔPDZb can bind to δ-catenin WT) (Fig. 4g,h). In accordance with previous reports showing that DHHC5 binds to PSD-95 via PDZ interactions^16^, DHHC5 ΔPDZb was unable to bind to PSD-95 (Fig. 4g,h). This demonstrates that DHHC5 binds to δ-catenin and PSD-95 through independent mechanisms.

Interestingly, the palmitoylation of δ-catenin and not its ability to bind to DHHC5 is sufficient for its translocation of δ-catenin to spines. Indeed, although overexpression of DHHC5 WT increased the recruitment of δ-catenin into spines, overexpression of DHHS5 that binds but does not palmitoylate δ-catenin^12^ does not (Fig. 4i,j). This is consistent with our observation that palmitoylation-deficient δ-catenin C960-1S is not recruited to spines following activity^12^. Together, this suggests that the transient association of DHHC5 and δ-catenin within dendritic shafts after activity is insufficient for its translocation to spines. Instead, we propose that palmitoylation of δ-catenin tethers it to vesicular membranes such as REs for its translocation into spines.

### Fyn phosphorylates and stabilizes DHHC5 at the cell surface

Although PSD-95 is not a substrate for DHHC5 (refs 16, 37), it is among its most frequently identified binding partners^16^. As DHHC5 and PSD-95 are transiently dissociated following activity, we hypothesized that PSD-95 binding stabilizes DHHC5 at synapses. PSD-95 is known to bind to the membrane-associated, Src-family tyrosine kinase Fyn^38^, recruiting it to the proximity of GluN2B–NMDARs to enhance Fyn binding and Fyn-mediated phosphorylation of GluN2B^38^,^39^. This results in enhanced stability of GluN2B–NMDARs at the synaptic membrane^40^. We therefore determined whether PSD-95 plays a similar role in the surface stabilization of DHHC5. Specifically, we determined whether PSD-95 stabilizes DHHC5 at the cell surface by recruiting Fyn, enhancing Fyn-mediated phosphorylation of DHHC5 and inhibiting the association of DHHC5 with endocytic proteins.

**Figure 5:**
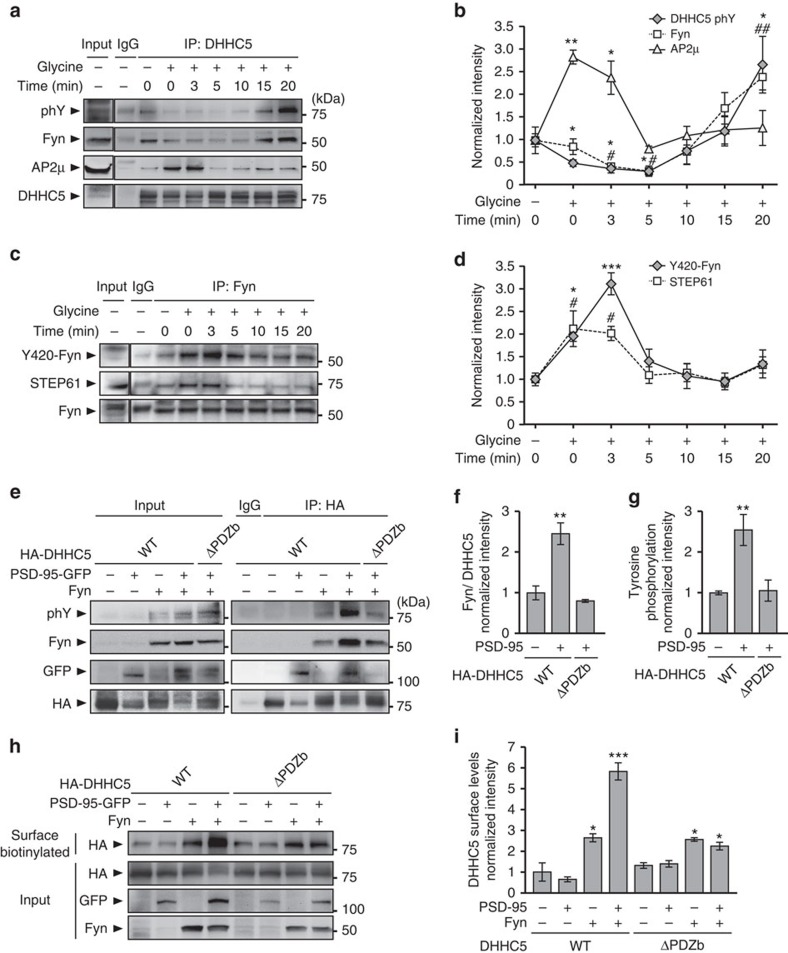
PSD-95 enhances the binding and tyrosine phosphorylation of DHHC5 by Fyn kinase. (**a**–**d**) Fourteen DIV hippocampal neurons were stimulated, lysed at the indicated time points, immunoprecipitated with anti- (**a**) DHHC5 or (**c**) Fyn and blots probed with the indicated antibodies. (**a**,**b**) DHHC5 tyrosine phosphorylation (phY; *P*=0.007, F_6,14_=7.749) and DHHC5/Fyn interactions (*P*<0.001, F_6,14_=11.06) are decreased transiently, whereas DHHC5/AP2μ interactions are increased transiently following cLTP (*P*=0.0076, F_6,14_=7.78). (**c**,**d**) Non-phosphoryated tyrosine 420 Fyn levels (*P*=0.0001, F_6,14_=11.24) and STEP61/Fyn interactions (*P*=0.004, F_6,14_=5.549) are transiently increased following cLTP. (**e**–**i**) HEK293T cells were transfected with the indicated HA-DHHC5, PSD-95-GFP or Fyn constructs for 36 h. (**e**,**f**) PSD-95 enhances Fyn/DHHC5 WT but not Fyn/DHHC5 ΔPDZb interactions (*P*=0.0014, F_2,6_=24.11). (**e**,**g**) PSD-95 enhances Fyn-mediated phosphorylation of DHHC5 WT but not DHHC5 ΔPDZb (*P*=0.0104, F_2,6_=10.75). (**h**,**i**) Lysates were biotinylated and immunoprecipitated with neutravidin-coated beads and blots probed with the indicated antibodies. (**i**) Normalized intensity of DHHC5 in the surface fraction (*P*<0.001, F_7,16_=43.23). (**b**,**d**,**f**,**g**,**i**) *n*=3 blots from 3 separate cultures. All graphs display mean±s.e.m. */^#^*P*<0.05, **/^##^*P*<0.01, ***P<0.001; one-way analysis of variance; Tukey's *post-hoc* test. Asterisks and cross-hatches above data points indicate significance relative to unstimulated controls for (**b**) DHHC5 phY or AP2μ, and Fyn or (**d**) Y420-Fyn and STEP61, respectively. (**a**,**c**,**e**,**h**) Five per cent of whole-cell lysates were loaded as inputs and (**a**,**c**) are from the same blots but with different exposure times. Full-length blots are presented in Supplementary Fig. 5.

We first demonstrated that endogenous DHHC5 binds to Fyn kinase in hippocampal neurons and is tyrosine phosphorylated (Fig. 5a,b and Supplementary Fig. 3a,b). As increased neuronal activity transiently decreases DHHC5 and PSD-95 interactions, we next examined the effects of activity on DHHC5 phosphorylation and its association with Fyn. DHHC5 tyrosine phosphorylation was significantly decreased 0–5 min after cLTP and DHHC5/Fyn interactions significantly decreased 3–5 min after cLTP stimulation. In conjunction with this, we observed an increase in DHHC5 association with the μ-subunit of the clathrin adaptor protein AP2 (AP2μ) 0–3 min after cLTP (Fig. 5a,b), in agreement with the rapid endocytosis of DHHC5 (Fig. 3a,d). Notably, DHHC5/Fyn interactions and Fyn tyrosine phosphorylation was much higher than basal levels 20 min after cLTP, indicating a more long-term change in the localization of these proteins after activity (Fig. 5a,b and Supplementary Fig. 3a,b).

To determine whether cLTP decreases Fyn activity as well as its association with DHHC5, we examined Fyn kinase activity as assessed by its autophosphorylation at tyrosine 420 (Y420). Indeed, autophosphorylation of this tyrosine domain, which resides within the activation loop of the Fyn kinase domain, maintains the protein in an active state^39^. The striatal enriched tyrosine phosphatase 61-kDa variant (STEP61) has been shown to decrease Fyn kinase activity by dephosphoylating Fyn Y420 (ref. 41). As STEP61 activity is increased following NMDAR stimulation^42^, we monitored STEP61/Fyn interactions as well as Fyn phosphorylation using an antibody that recognizes non-phosphorylated Y420 Fyn (Y420-Fyn). We observed a transient increase in STEP61/Fyn interactions and the fraction of non-phosphorylated Y420-Fyn 0–3 min following cLTP (Fig. 5c,d). Together, these results demonstrate that activity decreases the association of Fyn and DHHC5, and the phosphorylation of DHHC5 through STEP61-mediated dephosphorylation of Fyn, and the subsequent attenuation of Fyn activity. This primes DHHC5 for engaging with the endocytosis machinery.

To determine whether PSD-95 regulates Fyn/DHHC5 interactions, HEK293T cells were transfected with Fyn plus either DHHC5 WT or DHHC5 ΔPDZb in the presence or absence of PSD-95. Although Fyn binds to DHHC5 WT in the absence of PSD-95, this association is increased 2.5-fold in the presence of PSD-95 (Fig. 5e,f). PSD-95 did not enhance the association between Fyn and DHHC5 ΔPDZb, indicating that PSD-95 binds to DHHC5 in a PDZ-dependent manner, to increase DHHC5 association with Fyn (Fig. 5e,f). Interestingly, enhanced Fyn association was correlated with increased DHHC5 phosphorylation (Fig. 5e,f).

Expression of Fyn in HEK293T cells significantly increased the surface fraction of DHHC5 WT and DHHC5 ΔPDZb. Although expression of PSD-95 further increased the surface fraction of DHHC5 WT, it did not affect surface DHHC5 ΔPDZb levels (Fig. 5h,i). Together, these results show that the assembly of a PSD-95/Fyn/DHHC5 complex promotes DHHC5 phosphorylation and surface localization.

### Fyn inhibits the association of DHHC5 with endocytic proteins

**Figure 6:**
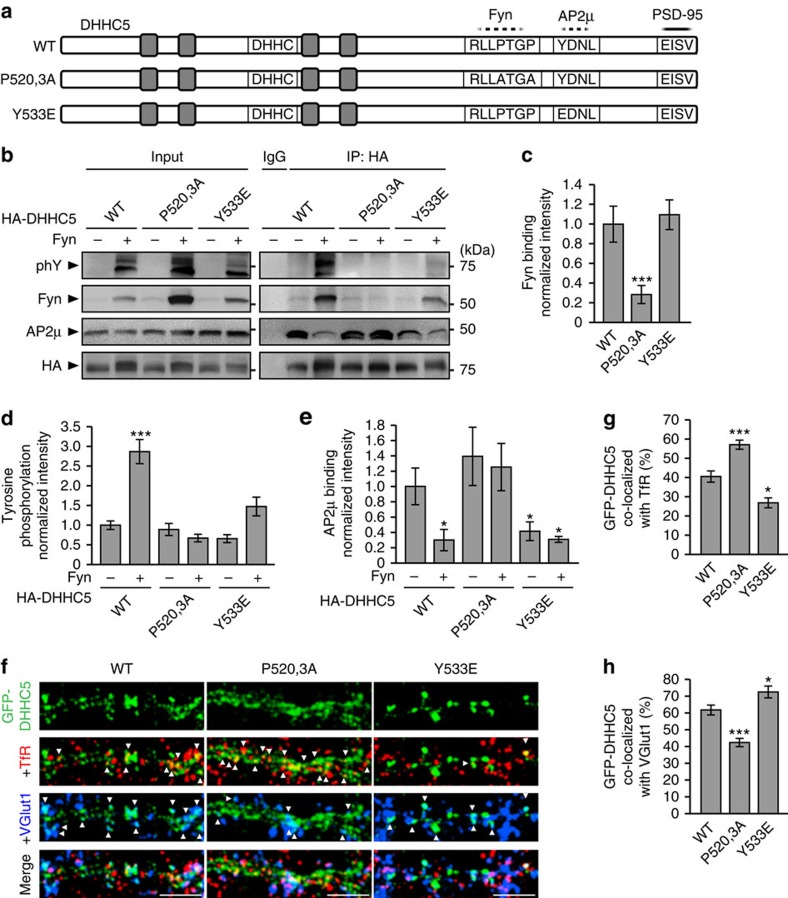
Phosphorylation of DHHC5 regulates its association with endocytic proteins and its subcellular localization. (**a**) Schematic depiction of DHHC5 constructs N-terminally tagged with GFP or HA (not shown here) and illustrating the approximate localization of transmembrane domains (grey boxes), the DHHC motif, a putative Fyn-binding site (dashed line; RLLPTGP), a putative AP2μ-binding site (dashed line; YDNL) and the PDZ-binding motif required for binding PSD-95 (solid line; EISV). (**b**–**e**) HEK293T cells were transfected with the indicated HA-DHHC5 and Fyn constructs for 36 h, lysates immunoprecipitated with an HA antibody and blots probed with the indicated antibodies. (**b**,**c**) Fyn binding of the DHHC5 P520,3A mutant is reduced, but not for for the Y533E mutant (*P*=0.0153, F_2,6_=9.07). (**b**,**d**) Fyn-mediated tyrosine phosphorylation is attenuated in DHHC5 P520,3A and Y533E mutants (*P*<0.001, F_5,12_=20.04). (**b**,**e**) Fyn decreases AP2μ association with DHHC5 WT, but not P520,3A or Y533E mutants (*P*=0.018, F_5,12_=6.29). *n*=3 blots from 3 separate cultures. (**f**) Confocal images of 14 DIV neurons transfected with the indicated GFP–DHHC5 construct and immunostained for TfR and VGluT1. Scale bar, 5 μm. (**g**) DHHC5 P520,3A increases and Y533E decreases co-localization with TfR (*P*<0.001, F_2,58_=24.05), and (**h**) decreases and increases co-localization with VGlut1, respectively (*P*<0.001, F_2,58_=23.5). *n*=22 (WT), 18 (P520,3A) and 21 (Y533E) cells from 3 cultures. Co-localized puncta are denoted by white arrowheads. All graphs display mean±s.e.m. *P<0.05, **P<0.01, ***P<0.001; one-way analysis of variance; Tukey's *post-hoc* test. (**b**) Five per cent of whole-cell lysates were loaded as inputs. Full-length blots are presented in Supplementary Fig. 5.

To further understand the mechanism by which Fyn stabilizes DHHC5, we sought to identify how DHHC5 binds to Fyn, the site of tyrosine phosphorylation and the function of DHHC5 phosphorylation. Fyn interacts with PSD-95 through its Src homology (SH)-2 domain^38^, leaving its SH3 domain available for additional protein interactions at the postsynaptic density^39^. Modular recognition sequences for SH3 domains predominantly consist of poly-proline tracts flanked by basic residues^43^,^44^,^45^. We identified a potential SH3-binding motif of Arg-Leu-Leu-Pro-Thr-Gly-Pro (RLLPTGP) within the carboxy-terminal domain of DHHC5 (residues 517–523; Fig. 6a) and predicted the core prolines 520 and 523 would be the most critical for SH3 binding^43^. To examine this, prolines 520 and 523 were mutated to alanines (P520,3A; Fig. 6a) and assayed their ability to bind Fyn in HEK293T cells (Fig. 6b,c). DHHC5 P520,3A did not bind Fyn and was not tyrosine phosphorylated in the presence of Fyn, indicating that Fyn binds directly to DHHC5 via SH3 interactions, and that this binding is required for Fyn-mediated phosphorylation (Fig. 6b,d).

Src family kinases commonly phosphorylate tyrosines within the immediate vicinity of their substrates' SH3-rocognition sequences^39^,^44^. Tyrosine 533 (Y533), 10aa from the Fyn binding site, is a highly predicted phosphorylation site (NetPhos 2.0; ref. 46) and has previously been identified as a phosphorylated residue^47^. Although mutating Y533 to the phospho-mimetic glutamic acid (Y533E) did not disrupt Fyn binding (Fig. 6b,c), it did inhibit Fyn-mediated tyrosine phosphorylation (Fig. 6b,d), demonstrating that Fyn phosphorylates DHHC5 at Y533.

Interestingly, Y533 lies within a canonical, tyrosine-based recognition motif for AP2μ (Tyr-Asp-Asn-Leu; YDNL)^48^,^49^,^50^ and phosphorylation of Y533 is predicted to inhibit AP2μ binding^50^,^51^,^52^. We demonstrated that the association of AP2μ and Y533E DHHC5 was significantly reduced compared with AP2μ/DHHC5 WT interactions and was similar to AP2μ/DHHC5 WT interactions in the presence of Fyn kinase (Fig. 6b,e). This demonstrates that Fyn-binding and Fyn-mediated phosphorylation of DHHC5 at Y533 inhibits the association of DHHC5 with endocytic proteins and enhances its stability at the membrane.

We next demonstrated that the binding of DHHC5 to Fyn or AP2μ mediates its localization at synapses or REs, respectively. GFP-DHHC5 WT (Fig. 6f,g), similar to endogenous DHHC5 (Fig. 3c), exhibited 40.71±2.95% co-localization with TfR and 61.84±2.93% co-localization with VGlut1 (a faithful marker of excitatory synapses; Figs 1c, 2a and 6f–h, and Supplementary Fig. 1a,b). Mutant DHHC5 that cannot bind to Fyn (P520,3A) co-localized more with TfR and less with VGlut1 (Fig. 6f–h). In contrast, mutant phospho-mimetic DHHC5 localized less with TfR and more with VGlut1 (Fig. 6f–h). Together, we show that phosphorylation of DHHC5 residue Y533 by Fyn kinase regulates the dynamic localization of DHHC5 between RE and synaptic compartments.

### Fyn and PSD-95 stabilize DHHC5 in spine heads

**Figure 7:**
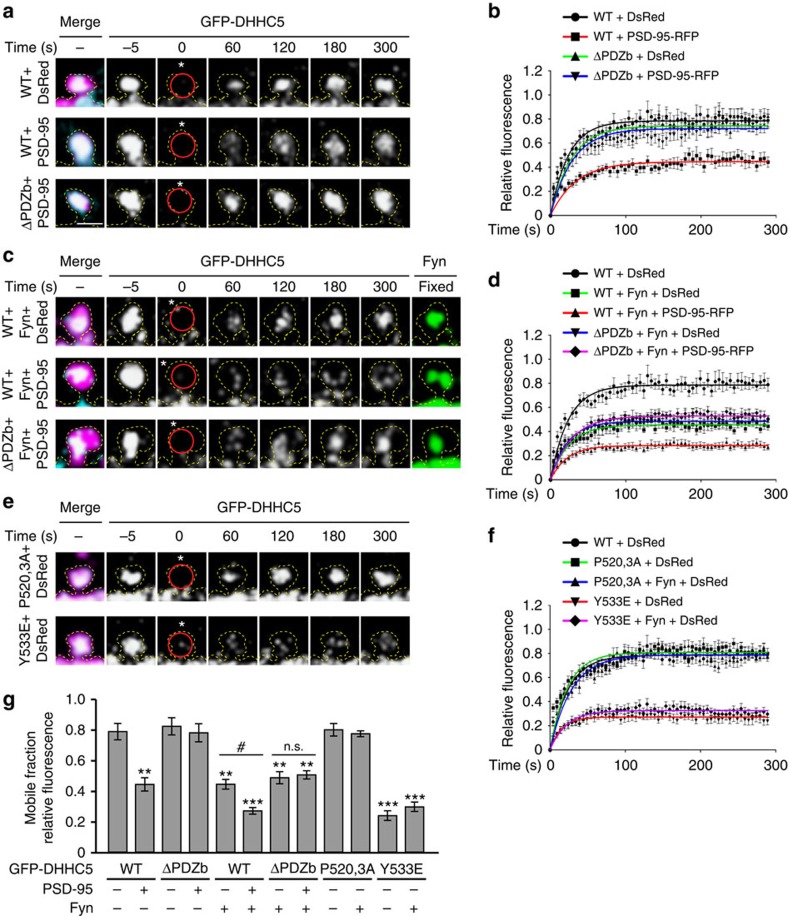
PSD-95 and Fyn control DHHC5 turnover in spine heads. (**a**,**c**,**e**) High-magnification confocal images of 14–16 DIV hippocampal neurons transfected with the indicated GFP–DHHC5, DsRed, PSD-95–RFP or Fyn constructs. GFP–DHHC5 fluorescence within a photobleached ROI (red circles) was analysed over 300 s (cells were initially photobleached at 0 s, white asterisks, within a 1-μm diameter ROI). Scale bar, 1 μm. (**b**,**d**,**f**) Relative fluorescence recovery of GFP–DHHC5. Solid lines represent single exponential fit. Points with error bars represent the mean±s.e.m. Statistical tests compare the plateau values from exponential fits±s.e.m. Neurons were obtained from three to five separate cultures. (**a**,**b**) Overexpression of PSD-95 significantly reduces the mobility of DHHC5 WT, but not ΔPDZb (*P*<0.001, F_3,77_=257.8; *n*=29 (DHHC5 WT+DsRed), 17 (WT+PSD-95-RFP), 20 (ΔPDZb+DsRed), 15 (ΔPDZb+PSD-95-RFP)). (**c**,**d**) Co-expression of Fyn and PSD-95 further decreases the mobile fraction of DHHC5 WT (*P*<0.001, F_4,94_=753.1; *n*=29 (WT+DsRed), 16 (WT+Fyn+DsRed), 19 (WT+Fyn+PSD-95), 16 (ΔPDZb+Fyn+DsRed), 19 (ΔPDZb+Fyn+PSD-95)). (**e**,**f**) Fyn does not impact the mobility of DHHC5 P520,3A nor Y533E (*P*<0.001, F_4,98_=1318; *n*=29 (WT+DsRed), 22 (P520,3A+DsRed), 17 (P520,3A+Fyn+DsRed), 18 (Y533E+DsRed), 17 (Y533E+Fyn+DsRed)). (**g**) The mobile fraction of GFP-DHHC5 (relative fluorescence fraction within the ROI at the 5-min time point normalized for photobleaching; mean±s.e.m.; *P*<0.001, F_11,213_=29.02; *n* values indicated above). *P<0.05, **P<0.01, ***P<0.001; one-way analysis of variance; Tukey's *post-hoc* test relative to control cells expressing WT+DsRed. n.s., not significant.

We next determined the role of PSD-95 and Fyn in regulating the turnover and mobility of DHHC5 in postsynaptic spine heads using fluorescence recovery after photobleaching. GFP–DHHC5 clusters within dendritic spines were identified and a region of interest (ROI) of 1-μm diameter was photobleached using a 405-nm laser. The fluorescence recovery of GFP–DHHC5 within the photobleached ROI was determined over 5 min of time-lapse imaging. In control neurons expressing GFP–DHHC5 and DsRed, the fluorescence recovery of GFP–DHHC5 plateaued at 78.8±5.4% (mean±s.e.m.; Fig. 7a,b,g), indicating that DHHC5 is highly mobile.

Expression of PSD-95 (Fig. 7a,b,g) or Fyn kinase (Fig. 7c,d,g) significantly stabilized DHHC5 WT within spines, as reflected in the overall reduction in GFP–DHHC5 fluorescence recovery. However, PSD-95 did not affect the mobility of DHHC5 ΔPDZb (Fig. 7a,b,g) and Fyn kinase did not affect the mobility of DHHC5 P520,3A (Fig. 7e–g), further confirming the requirement of the two domains for the formation of this tripartite complex. The phospho-mimetic DHHC5 Y533E mutant was significantly stabilized within spines compared with DHHC5 WT and was not further stabilized in the presence of Fyn (Fig. 7e–g). This further confirms that phosphorylation of DHHC5 by Fyn controls its stability at synaptic compartments. Thus, PSD-95 and Fyn promote synaptic surface stabilization and limit the endocytic cycling of DHHC5 by enhancing the phosphorylation of DHHC5 at Y533. Furthermore, disruption of AP2μ binding to DHHC5 in the Y533E mutant represses its endocytosis and cycling between cellular compartments.

### DHHC5 endocytosis is essential for AMPAR surface insertion

**Figure 8:**
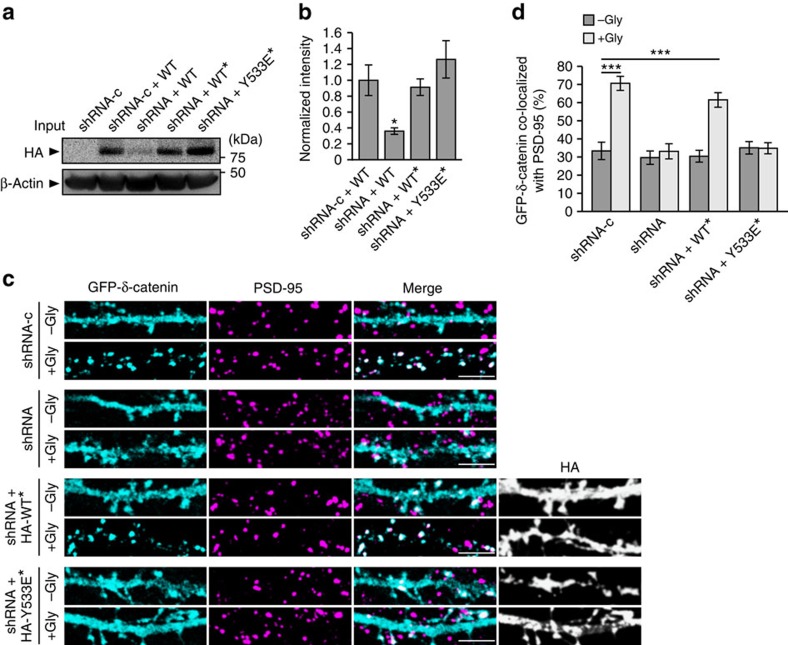
DHHC5 internalization is required for activity-induced δ-catenin trafficking. (**a**,**b**) HEK293T cells were transfected with control shRNA (shRNA-c) or shRNA against DHHC5 (shRNA) plus the indicated HA-DHHC5 constructs (*shRNA resistance) and blots probed with the indicated antibodies (*P*=0.024, F_3,8_=5.484, *n*=3 blots from 3 separate cultures; one-way analysis of variance (ANOVA)). (**c**) Confocal images of 14 DIV hippocampal neurons transfected with GFP–δ-catenin and the indicated shRNA and HA-DHHC5* constructs. Cells were stimulated with cLTP (+Gly) or control buffer lacking glycine (–Gly), fixed 20 min after stimulation and immunostained with the indicated antibodies. Scale bar, 5 μm. (**d**) The cLTP-induced increase in δ-catenin/PSD-95 co-localization is abolished in DHHC5 knockdown cells or those expressing the DHHC5 Y533E mutant (*P*<0.001, F_7,169_=17.44). The *n*-values, indicating neuron numbers from –Gly and +Gly cells, respectively, among three separate cultures are as follows: shRNA-c (23, 28), shRNA (17, 16), shRNA+WT* (22, 23) and shRNA+Y533E* (28, 22). Scale bar, 5 μm. Asterisks denote significance (**b**,**d**) among all groups relative to (**b**) shRNA-c+WT cells or (**d**) to –Gly shRNA-c cells. All graphs display mean±s.e.m. *P<0.05, ***P<0.001, one-way ANOVA, Tukey's *post-hoc* test. Full-length blots of **a** are presented in Supplementary Fig. 5.

We have previously demonstrated that δ-catenin palmitoylation is a key step in synapse strengthening and the stabilization of AMPARs at the synaptic membrane^12^. We therefore hypothesized that the cycling of DHHC5 from synaptic membranes to REs would regulate activity-induced recruitment of δ-catenin to synapses and/or stabilization of AMPARs at the synaptic membrane. To test this, we knocked down DHHC5 at 10 DIV using a previously validated DHHC5 short hairpin RNA (shRNA)^11^,^12^ (Fig. 8a,b) and examined the localization of δ-catenin at synapses, as demarcated by PSD-95 (Fig. 8c,d). cLTP mediates the recruitment of δ-catenin to PSD-95 clusters in control cells expressing shRNA-c but not in cells expressing DHHC5 shRNA (Fig. 8c,d) as seen previously^12^. Expression of DHHC5 WT* in knockdown cells restored cLTP-mediated trafficking of δ-catenin to synapses, whereas expression of DHHC5 Y533E* did not (Fig. 8c,d). This further demonstrates that the internalization and trafficking of DHHC5 to dendritic shafts is required for the recruitment of δ-catenin to synapses.

**Figure 9:**
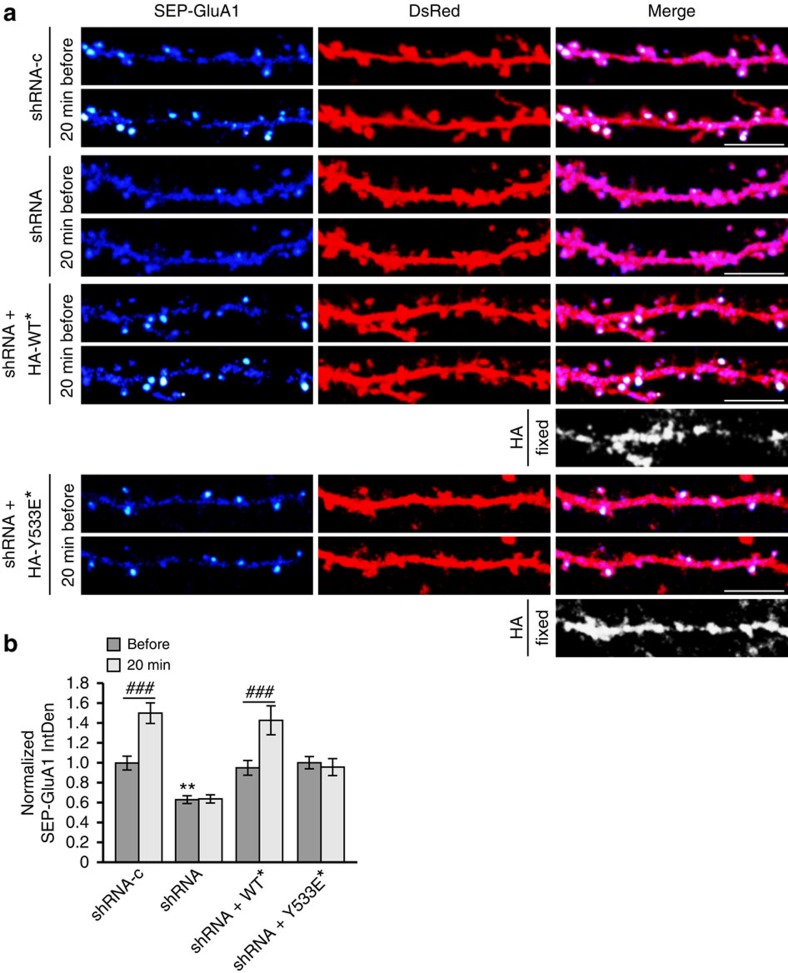
Activity-induced AMPAR surface insertion requires DHHC5 endocytic cycling. (**a**) Confocal images of 14–16 DIV neurons transfected with SEP-GluA1, DsRed and the indicated shRNA and HA-DHHC5* constructs (*shRNA-resistance). SEP-fluorescent puncta are pseudocoloured as heat maps. Cells were imaged before and 20 min after glycine stimulation. HA-DHHC5* expression was confirmed by *post-hoc* immunostaining for HA. Scale bar, 5 μm. (**b**) IntDen of SEP-GluA1 puncta in hippocampal neurons normalized to the same puncta before glycine treatment. The *n*-values, indicating neuron numbers from three separate cultures, and the *P*-values from paired *t*-tests are as follows: shRNA-c (22, *P*<0.001), shRNA (18, *P*=0.769), shRNA+WT* (18, *P*=0.0009) and shRNA+Y533E* (18, *P*=0.468). Knockdown of DHHC5 decreased basal SEP-GluA1 IntDen (*P*=0.0016, F_3,72_=5.633, one-way analysis of variance (ANOVA)). Asterisks denote significance between groups before cLTP and relative to shRNA-c, and cross-hatches denote significance within groups before and after cLTP. Graph displays mean±s.e.m. **P<0.01, one-way ANOVA, Tukey's *post hoc* test; ^###^*P*<0.001, paired *t*-test.

We next determined whether DHHC5 internalization is essential for activity-induced stabilization of AMPARs in the synaptic membrane. Surface GluA1 levels were measured using GluA1 tagged with super-ecliptic pHluorin (SEP-GluA1). Neurons expressing DHHC5 shRNA exhibited a significant reduction in the IntDen of SEP-GluA1 under basal conditions (Fig. 9a,b), in agreement with the requirement of DHHC5 for Grip1b palmitoylation and constitutive synaptic delivery of AMPARs^11^. This reduction in surface GluA1 was rescued in shRNA-expressing neurons co-transfected with DHHC5 WT* and Y533E*, indicating that localization of DHHC5 to the synaptic membrane is sufficient to maintain AMPARs at the membrane under basal conditions (Fig. 9a,b). This is consistent with a previous study demonstrating that localization of endogenous DHHC5 at the postsynaptic density constitutively delivers AMPARs to the synapse through Grip1b palmitoylation^11^.

**Figure 10:**
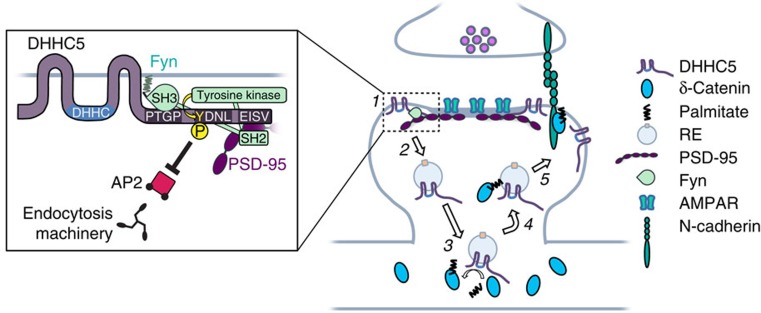
Model of activity-regulated trafficking of DHHC5. (1 and inset) Under basal conditions, DHHC5 is localized to the postsynaptic membrane in complex with PSD-95 and Fyn. PSD-95 binds DHHC5 through PDZ-dependent mechanisms. Fyn binds to the C-terminal poly-proline motif (PTGP) of DHHC5 through its SH3 domain and phosphorylates DHHC5 Y533 within the DHHC5 endocytic motif (YDNL). This inhibits the binding of the endocytic adaptor protein, AP2, to DHHC5. (**2**) Increased neuronal activity enhances STEP61-mediated dephosphorylation of Fyn and reduces its kinase activity (not shown), as well as decreases DHHC5 association with PSD-95 and Fyn, thereby enhancing DHHC5 internalization and trafficking to dendritic shafts on REs (2–3 min post stimulation). (**3**) DHHC5 associates with δ-catenin and palmitoylates it (3–10 min post stimulation). (**4**) DHHC5 and palmitoylated δ-catenin traffic back into spines (3–20 min post stimulation). (**5**) DHHC5 dissociates from δ-catenin and is reinserted into the synaptic membrane (10–20 min post stimulation). Significantly more DHHC5 is recruited to the synaptic membrane 20 min post stimulation compared with basal levels. δ-Catenin binds and stabilizes N-cadherin, leading to enhancements in synapse structure and increased membrane stabilization of AMPARs 20–60 min post stimulation. The association of δ-catenin with PSD-95 and Fyn, and δ-catenin tyrosine phosphorylation are also increased 20 min post stimulation (not shown), possibly linking cadherin–adhesion complexes with receptor-scaffold assemblies to coordinate synapse strengthening.

The IntDen of SEP-GluA1 was significantly increased 20 min after cLTP in shRNA-c control cells, relative to the same clusters before stimulation (Fig. 9a,b), consistent with previous reports^12^,^22^,^28^,^33^. This activity-induced increase in SEP-GluA1 clusters was abolished in shRNA-expressing neurons (Fig. 9a,b), in agreement with the requirement for δ-catenin palmitoylation in activity-induced surface localization of AMPARs^12^. DHHC5 WT*, but not Y533E*, rescued the knockdown phenotype (Fig. 9a,b), indicating the requirement of DHHC5 internalization for the activity-mediated stabilization of AMPAR at the synapse. Together, our results demonstrate that the internalization of DHHC5 is essential for activity-induced recruitment of δ-catenin into spines and the insertion and stabilization of AMPARs into the synaptic membrane. A model for our findings is shown in Fig. 10.

## Discussion

A number of key synaptic proteins including PSD-95 (ref. 8), δ-catenin^12^, gephyrin^53^, AKAP79/150 (ref. 22) and cdc42 (ref. 10) has been shown to be palmitoylated in an activity-dependent manner. This regulates their clustering and trafficking, thereby modulating the plasticity and function of synapses. However, to date the molecular mechanism(s) underlying activity-mediated palmitoylation of synaptic proteins remains largely unknown. Our study demonstrates that under basal conditions, PSD-95 and Fyn cooperatively stabilize DHHC5 at the synaptic membrane through Fyn-mediated phosphorylation of DHHC5 at tyrosine residue 533 and the subsequent inhibition of DHHC5 association with endocytic proteins (Fig. 10). Increased synaptic activity decreases the tyrosine kinase activity of Fyn and decreases Fyn/PSD-95 interactions with DHHC5, thereby enhancing the internalization of DHHC5 from the membrane and its trafficking to the dendritic shaft on REs. This activity-driven change in the subcellular localization of DHHC5 positions it closer to it substrate, δ-catenin, resulting in the association of this enzyme/substrate pair and the palmitoylation of δ-catenin. We postulate that palmitoylated δ-catenin is tethered to the RE membrane and is trafficked into synaptic spines together with DHHC5 on these endosomes. Upon delivery of δ-catenin to the synaptic membrane, it is able to associate with cadherin, stabilize surface cadherin and AMPARs, and increase synapse efficacy^12^. Together, this work demonstrates that synaptic activity can drive the trafficking of DHHC proteins between subcellular compartments and thereby alter their ability to associate with and palmitoylate downstream targets.

DHHC proteins are localized to multiple subcellular domains, with the vast majority restricted to various endomembrane compartments^29^. However, DHHC2, DHHC5, DHHC8 and DHHC14 constitute a small subset of PATs that have been shown to localize to the plasma membrane and which contain signalling motifs in their C-terminal tails that control their recruitment to the cell surface^8^,^15^. Further bioinformatic analysis of these PATS reveal tyrosine-based AP2μ-binding motifs (YxxΦ; Φ denoting any hydrophobic amino acid) at their C termini (Supplementary Fig. 4a–c), suggesting turnover of these surface proteins. Indeed, DHHC2 has been shown to traffic to the synaptic membrane in response to neuronal activity^8^,^9^ and its tyrosine-based endocytosis motif may play a role in its dynamic membrane localization (Supplementary Fig. 4a). Interestingly, sequence alignment of DHHC5 and DHHC8 revealed a conserved YDNL motif in close proximity to a poly-proline SH3-binding motif as well as di-leucine sites that can bind AP2 subunits^50^ (Supplementary Fig. 4b). The presence of tyrosine-based endocytosis signals in the C-terminal domains of these cell membrane-associated DHHCs suggests that the mechanism controlling the dynamic localization of DHHC5 in neurons may be conserved among multiple DHHCs. It is noteworthy that no endocytic signals are present in the C-terminal domain of DHHC3, a PAT known to localize to the somatic Golgi^8^ (Supplementary Fig. 4d).

Fyn and other members of the Src family of tyrosine kinases are widely expressed in the hippocampus where they play critical modulatory roles in synaptic protein trafficking and synapse plasticity^39^. Fyn is essential for the induction of LTP and the acquisition of contextual and spatial memories^54^,^55^. Fyn binds and phosphorylates the NMDAR subunit, GluN2B, at a tyrosine residue within an AP2μ-binding site, thereby blocking GluN2B association with endocytic proteins and stabilizing GluN2B at the synaptic membrane^40^. Fyn also interacts with PDZ-domain 3 of PSD-95 through its SH2 domain^38^, leaving its SH3 domain available for substrate binding and its tyrosine kinase domain in an active state^39^. Intriguingly, DHHC5 also interacts with PDZ-domain 3 of PSD-95 (ref. 16), positioning it in close proximity to the available SH3 domain of PSD-95-bound Fyn. Binding of Fyn by PSD-95 has been shown to enhance Fyn/GluN2B interactions and GluN2B phosphorylation^38^,^39^, consistent with our observations of the role of PSD-95 in regulating Fyn/DHHC5 interactions. Therefore, PSD-95 promotes DHHC5 surface localization by serving as a scaffold for the assembly of a PSD-95/Fyn/DHHC5 tripartite complex and the subsequent phosphorylation of DHHC5 by Fyn.

How does enhanced activity dissolve the DHHC5/Fyn/PSD-95 complex? Previous work has shown that activity-induced influx of Ca^2+^ into spines through NMDARs results in an increase in the binding of Ca^2+^/calmodulin to the amino terminus of PSD-95 (ref. 56). This in turn inhibits the palmitoylation of PSD-95 and decreases its stability at the postsynaptic density^56^. Indeed, this results in the transient removal of PSD-95 from spine heads 1–2 min after LTP stimulation, followed by its reinsertion into spines 5 min post stimulation^57^. We predict that activity-induced internalization of PSD-95 results in the dissolution of PSD-95/Fyn complexes at the synapse, which in turn destabilizes the membrane localization of other synaptic proteins. Previous work has shown that neuronal activity results in the dissociation of the Fyn/PSD-95 complex from GluN2B-containing NMDARs^58^. This enhances the binding of endocytic proteins to GluN2B and results in the internalization of NMDARs^40^,^58^. This is consistent with what we have observed for PSD-95, Fyn and DHHC5, highlighting mechanistic redundancies in activity-mediated regulation of synaptic proteins at the membrane. Together, we suggest that activity-induced influx of Ca^2+^ into spines may destabilize and remove PSD-95 from postsynaptic compartments, disassemble DHHC5/Fyn/PSD-95 complexes and result in the dephosphorylation and internalization of DHHC5.

The findings of this study demonstrate that synaptic activity can drive the trafficking of DHHC proteins between subcellular compartments and thereby alter their ability to associate with and differentially palmitoylate multiple downstream targets in various cellular regions. Palmitoylated Grip1b and δ-catenin, both DHHC5 substrates, have distinct but cooperative roles in regulating the surface localization of AMPARs^11^,^12^. Although the palmitoylation of δ-catenin has been shown to be activity dependent^12^, Grip1b palmitoylation is not^8^. Here we suggest that the dynamic localization of DHHC5 provides an explanation for this apparent distinction between its two substrates. Under basal conditions, Grip1b is enriched in the postsynaptic compartment in the proximity of DHHC5 (ref. 11) and its palmitoylation by DHHC5 targets it to dendritic REs where it continually cycles between synaptic compartments and maintains AMPAR delivery^11^,^59^. Consequently, shaft-localized δ-catenin is sequestered from DHHC5 under basal conditions and exhibits low palmitoylation levels^12^. We propose that membrane-associated DHHC5 constitutively palmitoylates Grip1b to maintain synaptic AMPAR turnover, whereas enhanced activity results in the internalization of DHHC5, the palmitoylation and trafficking of δ-catenin into spines and the subsequent recruitment and stabilization of GluA1 containing AMPARs at the membrane.

Several forms of synaptic plasticity involve the insertion or removal of proteins from postsynaptic compartments^1^,^3^,^24^. Although cLTP has been shown to mediate a relatively long-term recruitment^3^ or expulsion^24^ of proteins from postsynaptic compartments, other proteins such as PSD-95 and DHHC5 display more transient changes. Indeed, both PSD-95 and DHHC5 are removed from spines 1–2 min after stimulation and then trafficked back into spines 5 min after stimulation^57^. Our work demonstrates that DHHC5 is transported on REs. Interestingly, the trafficking of REs and their cargoes in and out of spine heads significantly increases following LTP^1^,^22^,^28^. This activity-induced RE trafficking delivers AMPARs to the synaptic membrane 1–3 min following cLTP^28^,^34^ and mediates AMPAR endocytosis 5 min following cLTD^32^,^60^. Such dynamic activity-dependent molecular reorganization of the postsynaptic compartment via RE transport illustrates the complex choreography of protein trafficking required for synapse plasticity to occur.

Interestingly, mutations in the genes encoding DHHC5 and its homologue DHHC8 have repeatedly been linked to schizophrenia and other neuropsychiatric disorders^17^,^18^,^19^,^61^,^62^,^63^. Furthermore, both DHHC5 (ref. 16) and δ-catenin^64^,^65^ have been implicated in higher-order brain functions such as learning and memory, raising the possibility that aberrant functioning of this enzyme–substrate pair may disrupt synapse plasticity and contribute to the pathology underlying these conditions. Our study demonstrates that the precise activity-regulated trafficking of DHHC5 is required for the synaptic targeting of δ-catenin and may be an essential component of synapse function and learning and memory.

## Methods

### Antibodies and complementary DNA constructs

Primary antibodies used were as follows: δ-catenin (1:500 western blot (WB), 5 μg immunoprecipitation (IP); BD Transduction Laboratories 611536), N-cadherin (1:500; BD Transduction Laboratories 610921), PSD-95 for immunocytochemistry (ICC; 1:500; Abcam ab2723), PSD-95 for IP and WB (5 μg, 1:500; Calbiochem CP35), Gephyrin (1:500; Synaptic Systems 147 011), GFP for IP (10 μl; Synaptic Systems 132 002), GFP for WB (1:1,000; Roche 11814460001), DHHC5 (1:500 ICC, 1:1,000 WB, 1 μg IP; Sigma Prestige HPA014670), TfR (1:500; Millipore GR09L), VPS-35 (1:500; Abnova H000055737-M02), GluA1 (1:1,000; Millipore 05-855R), haemagglutinin (HA) for ICC (1:500; Cell Signaling Technology C29F4), HA for IP and WB (5 μg, 1:500; Covance MMS-101P), VGlut1 (1:500; Millipore AB5905), Fyn for WB and ICC (1:250, 1:500; BD Transduction Laboratories 610163), Fyn for IP (5 μg; Life Technologies MA5-13134), non-phosphorylated Y420 Fyn (1:1,000; Cell Signaling Technologies 2102S), STEP61 (1:1,000; Life Technologies MA1-16746), phospho-tyrosine (phY; 1:1,000 WB, 5 μg IP; Millipore 4G10/05-321), AP2μ (1:500; Thermo Scientific PA5-20745) and β-actin (1:1,000; Sigma A1978). Secondary antibodies used were as follows: IgG-horseradish peroxidase (HRP; 1:5,000; BioRad mouse 170-6516 and rabbit 170-6515), IgY-HRP (1:5,000; LifeSpan BioSciences LS-C86499), Streptavidin-HRP (1:5,000; Thermo Scientific 21126) and Alexa-Fluor 568 goat anti-mouse and 633 goat anti-rabbit (1:1,000; Life Technologies A-11004 and A-21070, respectively).

GFP–δ-catenin and RFP–δ-catenin were generated as previously described^12^. TfR-mCherry was a kind gift from Dr Michael Silverman (Simon Fraser University, Vancouver, BC). HA-DHHC5, HA-DHHS5, DHHC5-targeting and control shRNAs were kind gifts from Dr Richard Huganir (Johns Hopkins University, Balitmore, MD). The GFP–DHHC5 construct was generated by PCR of DHHC5 from HA-DHHC5 (accession number: NM_00139388) using an EcoRI-tagged forward primer (5′-CCGGCGAATTCTATGCCCGCAGAGTCTG-3′) and a BamHI-tagged reverse primer (5′-CGAGATTTCTGTGTGAGGATCCCCGGC-3′), and pasted into pEGFP-C1 using EcoRI and BamHI sites. HA-DHHC5 that was resistant to shRNA (denoted by *) was generated as previously described^12^, and P520,3A and Y533E point mutations were generated by site-directed mutagenesis using the GFP-DHHC5, HA-DHHC5 and HA-DHHC5* constructs as templates. Fyn kinase was generated by Dr Filippo Giancotti (Sloan Kettering Institute, New York, NY) and obtained from Addgene (number 16032), and SEP-GluA1 was generated by Dr Roberto Malinow (University of California, San Diego) and also obtained from Addgene (number 24000).

### Cell cultures

#### Primary hippocampal neurons

Hippocampi from embryonic day 18 (E18) Sprague–Dawley rats of either sex were prepared as previously described^66^ and plated at a density of 130 cells per mm^2^. A NeuroCult SM1 supplement (Stem Cell Technologies 05711) was used in the place of B27 in maintenance media. Neurons were transfected with Lipofectamine 2000 (Invitrogen) at 9–10 DIV, according to the manufacturer's recommendations, and used for experiments at 12–16 DIV.

*HEK cells*. HEK293T cells were transfected using polyethylenimine (Sigma) as previously described^12^, in a 3:1 ratio with the total plasmid DNA to be transfected. 293T cells were transfected at 70%–80% confluency and incubated for 24–48 h before harvesting for biochemistry.

### Neuronal activation

Neuronal activity was modified using previously described cLTP or chemical LTD protocols^1^,^2^,^12^,^26^. Briefly, maintenance media was replaced with an extracellular recording solution containing the following: 125 mM NaCl, 5 mM KCl, 2 mM CaCl_2_, 0 mM MgCl_2_, 5 mM HEPES, 33 mM D-glucose and supplemented with 0.5 μM tetrodotoxin, 20 μM Bicuculline pH 7.3, 290 mOsm l^−1^, for 10–15 min. For cLTP, this media was supplemented with 200 μM glycine or 200 μM glycine plus 50 μM DL-2-Amino-5-phosphonopentanoic acid (AP5) (Sigma) for 3 min. For cLTD, the media was supplemented with 20 μM NMDA (Sigma) plus 10 μM glycine for 3 min. The solution was then replaced with fresh extracellular recording solution (containing 0 mM MgCl_2_ for LTP or 2 mM MgCl_2_ for LTD) for the indicated times before experimentation. Cells were continually maintained at 37 °C for the duration of activity stimulation.

### ABE assay

ABE assays were performed as previously described^21^. Briefly, cells were lysed in lysis buffer supplemented with 50 mM N-ethylmaleimide and target proteins immobilized overnight on protein A/G-conjugated sepharose beads by IP at 4 °C. Immunocomplexes were then washed and treated with lysis buffer at pH 7.2 and supplemented with 1 M hydroxylamine (NH_2_OH) for 1 h at room temperature (RT), then washed and treated with lysis buffer at pH 6.2 and supplemented with 1 μM biotin-BMCC (Thermo Scientific) for 1 h at 4 °C. Target proteins were then eluted in a 2 × loading buffer containing 5 mM dithiothreitol and palmitoylated proteins analysed by immunoblotting.

### Biotinylation assay

Biotinylation of all surface proteins was performed as previously described^67^. Briefly, cells were washed in ice-cold PBS supplemented with 1 mM MgCl_2_ and 0.1 mM CaCl_2_ (PBS-CM) and treated with PBS-CM supplemented with 0.5 mg ml^−1^ of Sulfo-NHS-SS-biotin (Thermo Scientific) for 30 min at 4 °C for the duration of the reaction. Cells were then washed once with PBS-CM, then twice with PBS-CM supplemented with 20 mM glycine for 7 min each at 4 °C. Cells were then lysed in lysis buffer of PBS containing 1% IGEPAL CA-630 detergent at 4 °C and supplemented with the inhibitors described above. Surface proteins were then immobilized on Neutravidin agarose beads (Thermo Scientific) overnight at 4 °C, washed five times in standard lysis buffer containing 137 mM NaCl (or titrated to higher concentrations, where indicated), eluted in SDS sample buffer containing 100 mM dithiothreitol (or in the absence of any reducing agents, where indicated) and analysed by immunoblotting. Fifty per cent of whole-cell lysates (25–50 μg) were loaded as input samples.

### Immunoprecipitation

IP assays were performed as previously described^12^. Briefly, lysates were incubated overnight at 4 °C with the indicated antibody. Fifty microlitres of protein A/G–Sepharose (GE Healthcare, Chicago, IL) was added to the lysates and the bead-bound immunocomplexes were recovered after 2 h, washed four times with lysis buffer, solubilized with loading buffer, separated by SDS–PAGE and analysed by immunoblotting with the indicated antibodies. Five per cent of whole-cell lysates were loaded as input samples.

### Western blot analysis

Western blotting was performed as previously described^12^. Brain tissue, primary hippocampal neurons and 293T cells were homogenized in an ice-cold lysis buffer containing 1% IGEPAL CA-630 (Sigma), 0.5% Triton X-100, 50 mM Tris-HCl pH 7.5, 150 mM NaCl, 10% glycerol and supplemented with phenylmethanesulfonyl fluoride solution and a protease inhibitor cocktail with EDTA (Roche). Whole-cell lysates were subjected to further mechanical disruption by passage through a 26-gauge syringe 5–6 times. Lyates were cleared by centrifugation at 16,100*g* for 30 min at 4 °C and the solubilized fraction of protein was used for all biochemical experiments. Lysis buffer used in experiments requiring detection of phospho-tyrosine was additionally supplemented with a phosphatase inhibitor cocktail (PhosSTOP, Roche; 04906845001). Proteins were separated by SDS–PAGE, analysed by immunoblotting with the indicated antibodies and visualized using enhanced chemiluminescence (Pierce Biotechnology) on a Bio-Rad Versadoc 4000 (Bio-Rad Laboratories). Blots were quantified using Image J software. Full-length blots with molecular weight markers, denoting kDa, are shown in Supplementary Fig. 5.

### Immunocytochemistry

Immunocytochemistry experiments were performed as previously reported^12^. Briefly, cells were fixed in a 4% paraformaldehyde/sucrose solution pre-warmed to 37 °C for 10 min, then washed in PBS, treated with 0.1% Triton X-100/PBS for 10 min at RT, washed again with PBS and then blocked with 10% goat serum (GS)/PBS for 1 h at RT. Following blocking, cells were incubated with primary antibodies in 0.1% GS/PBS overnight at 4 °C. Subsequently, cells were washed in PBS three times for 10 min each, incubated in secondary antibodies in 0.1% GS/PBS for 1 h at RT, washed again in PBS three times and mounted on microscope slides in Prolong Gold (Molecular Probes).

### Confocal imaging

All neurons were imaged using an Olympus Fluoview 1000 confocal microscope (× 60/1.4 Oil Plan-Apochromat). Identical acquisition parameters were used for all cells across all separate cultures within an experiment. For time-lapse imaging of GFP–DHHC5 or SEP-GluA1, a ROI along a primary dendrite of a transfected cell within 100 μm from the cell body was chosen and imaged before, and again after the 3 min of cLTP/cLTD at the indicated time points. Dendritic spines were defined as any protrusion between 0.5 and 10 μm in length emanating from the dendritic shaft within the proximal 100 μm from the cell body. Confocal images shown in the figures were subjected to a 1 pixel Gaussian blur. The levels and contrast of confocal images were moderately adjusted in Photoshop CS6 software (Adobe Systems, Inc.) using scientifically accepted procedures.

### Fluorescence recovery after photobleaching

Dendritic spines within 100 μm of the cell body were imaged every 5 s for 5 min before and after photobleaching. Spines were identified using DsRed or PSD-95-RFP. A 1-μm circular ROI was photobleached within a spine head using the tornado function within Fluoview software (Olympus). The fluorescence of GFP–DHHC5 in the photobleached ROI was quantified over time using Fluoview software. The recovery of fluorescence intensity (*R*) was determined by normalizing the intensity at a specific time (*F*_t_), using the formula: *R*=(*F*_t_−*F*_0_)/(*F*_i_−*F*_0_) where (*F*_0_) represents the fluorescence at the time of photobleaching and (*F*_i_) the fluorescence before bleaching.

Fluorescence recovery data was collected in Prism software (GraphPad), analysed and fit to a single exponential model, which generated plateau values for the mean *R*-value among each group of cells. Plateau values±s.e.m. were statistically compared in Prism software.

### Image analysis and quantification

Confocal images for a particular experiment were subjectively thresholded using ImageJ software and the same threshold was used for all images obtained for a single experiment, throughout the experimental analysis. For live time-lapse imaging experiments, the same threshold was applied to the image acquired before stimulation and to all the images acquired afterwards at the indicated time points. Puncta were defined as a thresholded fluorescence cluster with an area between 0.05 and 3 μm^2^. Puncta area and IntDen (the product of area and mean grey value) were then determined using ImageJ. An Image J co-localization plugin was used to assess co-localization between different channels^12^ (http://rsb.info.nih.gov/ij/plugins/colocalization.html). Points of co-localization were defined as regions of >4 pixels in size, with >50 intensity ratio between the two channels.

For analysis of GFP–DHHC5 fluorescence within spines, a circular ROI was drawn around identified spines and the IntDen measured before and after stimulation.

The IntDen of SEP-GluA1 puncta was determined in Image J. Only SEP-fluorescent puncta that were present along dendrites before activity induction were considered for analysis.

For analysis of co-localization specifically within spines and dendrites, a mask was generated in Photoshop CS6 that outline fluorescence in spines and shafts. Using the paintbrush tool, the dendrite was manually highlighted, separating spines and dendrites, and enabling the generation of spine-only or dendrite-only masks. Specific masks were then applied to all channels to selectively analyse co-localization within spines or dendrites. Co-localization analysis was then done as described above.

To generate the spine contours shown in the images in Fig. 2e,g in the absence of a cellular volume filler, we adhered to previously described experimental procedures^28^,^33^. Briefly, a maximum intensity projection of the RFP–δ-catenin fluorescence intensity at each of the given imaging time points was generated in ImageJ. A thresholded mask of cell's outline was then created using the ImageJ magic wand tool. At high magnification in Photoshop CS6 software, the contours generated by the ImageJ magic wand tool were traced using the Photoshop pencil tool and then overlaid onto the image panels in Fig. 2e,g.

### Statistical analysis

All data values are expressed as mean±s.e.m. For all imaging experiments, ‘*n*' refers to the number of cells used per condition, over at least three separate cultures, with the exception of the analysis performed in Fig. 2c, where ‘*n*' refers to the number of spines and is specified within the figure legends. Our sample sizes are similar to those reported in the literature^9^,^11^,^12^. Data collection and analysis were not performed blind to the conditions of the experiment and were not acquired with any specific randomization procedure. However, cells were assigned to experimental groups and analysis was performed with absolutely no bias, and by different experimenters. All data were analysed in Prism software (GraphPad Software, Inc.) and met the assumption of normality by using a D'Agostino and Pearson omnibus normality test in Prism, with the exception of all biochemical data and some live-imaging experiments in which the ‘*n*' values were too small, and so a normal distribution was assumed and not formally tested. Statistical significance was determined by Student's *t*-test, one-way analysis of variance or repeated-measures one-way analysis of variance with *post-hoc* tests using Prism, where indicated. Statistical significance was assumed when *P*<0.05. In all figures, *P<0.05, **P<0.01 and ***P<0.001 as determined in Prism software. All figures were generated using Illustrator CS6 software (Adobe Systems, Inc.).

## Additional information

**How to cite this article:** Brigidi, G. S. *et al*. Activity-regulated trafficking of the palmitoyl-acyl transferase DHHC5. *Nat. Commun*. 6:8200 doi: 10.1038/ncomms9200 (2015).

## Supplementary Material

Supplementary Information Supplementary Figures 1-5

## References

[b1] Park M. . Plasticity-induced growth of dendritic spines by exocytic trafficking from recycling endosomes. Neuron 52, 817–830 (2006). 17145503 10.1016/j.neuron.2006.09.040 PMC1899130

[b2] Lu W. . Activation of synaptic NMDA receptors induces membrane insertion of new AMPA receptors and LTP in cultured hippocampal neurons. Neuron 29, 243–254 (2001). 11182095 10.1016/s0896-6273(01)00194-5

[b3] Bosch M. . Structural and molecular remodeling of dendritic spine substructures during long-term potentiation. Neuron 82, 444–459 (2014). 24742465 10.1016/j.neuron.2014.03.021 PMC4281348

[b4] Fu M., Yu X., Lu J. & Zuo Y. Repetitive motor learning induces coordinated formation of clustered dendritic spines *in vivo*. Nature 483, 92–95 (2012). 22343892 10.1038/nature10844 PMC3292711

[b5] Lai C. S., Franke T. F. & Gan W. B. Opposite effects of fear conditioning and extinction on dendritic spine remodelling. Nature 483, 87–91 (2012). 22343895 10.1038/nature10792

[b6] Iwanaga T., Tsutsumi R., Noritake J., Fukata Y. & Fukata M. Dynamic protein palmitoylation in cellular signaling. Prog. Lipid Res. 48, 117–127 (2009). 19233228 10.1016/j.plipres.2009.02.001

[b7] Fukata Y. & Fukata M. Protein palmitoylation in neuronal development and synaptic plasticity. Nat. Rev. Neurosci. 11, 161–175 (2010). 20168314 10.1038/nrn2788

[b8] Noritake J. . Mobile DHHC palmitoylating enzyme mediates activity-sensitive synaptic targeting of PSD-95. J. Cell Biol. 186, 147–160 (2009). 19596852 10.1083/jcb.200903101 PMC2712995

[b9] Fukata Y. . Local palmitoylation cycles define activity-regulated postsynaptic subdomains. J. Cell Biol. 202, 145–161 (2013). 23836932 10.1083/jcb.201302071 PMC3704990

[b10] Kang R. . Neural palmitoyl-proteomics reveals dynamic synaptic palmitoylation. Nature 456, 904–909 (2008). 19092927 10.1038/nature07605 PMC2610860

[b11] Thomas G. M., Hayashi T., Chiu S. L., Chen C. M. & Huganir R. L. Palmitoylation by DHHC5/8 targets GRIP1 to dendritic endosomes to regulate AMPA-R trafficking. Neuron 73, 482–496 (2012). 22325201 10.1016/j.neuron.2011.11.021 PMC3345505

[b12] Brigidi G. S. . Palmitoylation of delta-catenin by DHHC5 mediates activity-induced synapse plasticity. Nat. Neurosci. 17, 522–532 (2014). 24562000 10.1038/nn.3657 PMC5025286

[b13] Li Y., Martin B. R., Cravatt B. F. & Hofmann S. L. DHHC5 protein palmitoylates flotillin-2 and is rapidly degraded on induction of neuronal differentiation in cultured cells. J. Biol. Chem. 287, 523–530 (2012). 22081607 10.1074/jbc.M111.306183 PMC3249106

[b14] Kokkola T. . Somatostatin receptor 5 is palmitoylated by the interacting ZDHHC5 palmitoyltransferase. FEBS Lett. 585, 2665–2670 (2011). 21820437 10.1016/j.febslet.2011.07.028

[b15] He M., Abdi K. M. & Bennett V. Ankyrin-G palmitoylation and betaII-spectrin binding to phosphoinositide lipids drive lateral membrane assembly. J. Cell Biol. 206, 273–288 (2014). 25049274 10.1083/jcb.201401016 PMC4107783

[b16] Li Y. . DHHC5 interacts with PDZ domain 3 of post-synaptic density-95 (PSD-95) protein and plays a role in learning and memory. J. Biol. Chem. 285, 13022–13031 (2010). 20178993 10.1074/jbc.M109.079426 PMC2857114

[b17] Fallin M. D. . Genomewide linkage scan for bipolar-disorder susceptibility loci among Ashkenazi Jewish families. Am. J. Hum. Genet. 75, 204–219 (2004). 15208783 10.1086/422474 PMC1216055

[b18] Schizophrenia Working Group of the Psychiatric Genomics, C. Biological insights from 108 schizophrenia-associated genetic loci. Nature 511, 421–427 (2014). 25056061 10.1038/nature13595 PMC4112379

[b19] Fromer M. . *De novo* mutations in schizophrenia implicate synaptic networks. Nature 506, 179–184 (2014). 24463507 10.1038/nature12929 PMC4237002

[b20] Ohno Y. . Analysis of substrate specificity of human DHHC protein acyltransferases using a yeast expression system. Mol. Biol. Cell 23, 4543–4551 (2012). 23034182 10.1091/mbc.E12-05-0336 PMC3510016

[b21] Brigidi G. S. & Bamji S. X. Detection of protein palmitoylation in cultured hippocampal neurons by immunoprecipitation and acyl-biotin exchange (ABE). J. Vis. Exp 72, 50031 (2013). 23438969 10.3791/50031 PMC3605617

[b22] Keith D. J. . Palmitoylation of A-kinase anchoring protein 79/150 regulates dendritic endosomal targeting and synaptic plasticity mechanisms. J. Neurosci. 32, 7119–7136 (2012). 22623657 10.1523/JNEUROSCI.0784-12.2012 PMC3367663

[b23] Jurado S. . LTP requires a unique postsynaptic SNARE fusion machinery. Neuron 77, 542–558 (2013). 23395379 10.1016/j.neuron.2012.11.029 PMC3569727

[b24] Araki Y., Zeng M., Zhang M. & Huganir R. L. Rapid dispersion of SynGAP from synaptic spines triggers AMPA receptor insertion and spine enlargement during LTP. Neuron 85, 173–189 (2015). 25569349 10.1016/j.neuron.2014.12.023 PMC4428669

[b25] Musleh W., Bi X., Tocco G., Yaghoubi S. & Baudry M. Glycine-induced long-term potentiation is associated with structural and functional modifications of alpha-amino-3-hydroxyl-5-methyl-4-isoxazolepropionic acid receptors. Proc. Natl Acad. Sci. USA 94, 9451–9456 (1997). 9256503 10.1073/pnas.94.17.9451 PMC23219

[b26] Li D. . SAP97 directs NMDA receptor spine targeting and synaptic plasticity. J. Physiol. 589, 4491–4510 (2011). 21768261 10.1113/jphysiol.2011.215566 PMC3208220

[b27] Lee H. K., Kameyama K., Huganir R. L. & Bear M. F. NMDA induces long-term synaptic depression and dephosphorylation of the GluR1 subunit of AMPA receptors in hippocampus. Neuron 21, 1151–1162 (1998). 9856470 10.1016/s0896-6273(00)80632-7

[b28] Wang Z. . Myosin Vb mobilizes recycling endosomes and AMPA receptors for postsynaptic plasticity. Cell 135, 535–548 (2008). 18984164 10.1016/j.cell.2008.09.057 PMC2585749

[b29] Ohno Y., Kihara A., Sano T. & Igarashi Y. Intracellular localization and tissue-specific distribution of human and yeast DHHC cysteine-rich domain-containing proteins. Biochim. Biophys. Acta 1761, 474–483 (2006). 16647879 10.1016/j.bbalip.2006.03.010

[b30] Greaves J., Carmichael J. A. & Chamberlain L. H. The palmitoyl transferase DHHC2 targets a dynamic membrane cycling pathway: regulation by a C-terminal domain. Mol. Biol. Cell 22, 1887–1895 (2011). 21471008 10.1091/mbc.E10-11-0924 PMC3103404

[b31] Jaafari N., Henley J. M. & Hanley J. G. PICK1 mediates transient synaptic expression of GluA2-lacking AMPA receptors during glycine-induced AMPA receptor trafficking. J. Neurosci. 34, 11618–11630 (2012). 22915106 10.1523/JNEUROSCI.5068-11.2012 PMC6703756

[b32] Beattie E. C. . Regulation of AMPA receptor endocytosis by a signaling mechanism shared with LTD. Nat. Neurosci. 3, 1291–1300 (2000). 11100150 10.1038/81823

[b33] Kennedy M. J., Davison I. G., Robinson C. G. & Ehlers M. D. Syntaxin-4 defines a domain for activity-dependent exocytosis in dendritic spines. Cell 141, 524–535 (2010). 20434989 10.1016/j.cell.2010.02.042 PMC2874581

[b34] Petrini E. M. . Endocytic trafficking and recycling maintain a pool of mobile surface AMPA receptors required for synaptic potentiation. Neuron 63, 92–105 (2009). 19607795 10.1016/j.neuron.2009.05.025 PMC2847611

[b35] Tai C. Y., Mysore S. P., Chiu C. & Schuman E. M. Activity-regulated N-cadherin endocytosis. Neuron 54, 771–785 (2007). 17553425 10.1016/j.neuron.2007.05.013

[b36] Munsie L. N. . Retromer-dependent neurotransmitter receptor trafficking to synapses is altered by the Parkinson's disease VPS35 mutation p.D620N. Hum. Mol. Genet. 24, 1691–1703 (2014). 25416282 10.1093/hmg/ddu582

[b37] Fukata M., Fukata Y., Adesnik H., Nicoll R. A. & Bredt D. S. Identification of PSD-95 palmitoylating enzymes. Neuron 44, 987–996 (2004). 15603741 10.1016/j.neuron.2004.12.005

[b38] Tezuka T., Umemori H., Akiyama T., Nakanishi S. & Yamamoto T. PSD-95 promotes Fyn-mediated tyrosine phosphorylation of the N-methyl-D-aspartate receptor subunit NR2A. Proc. Natl Acad. Sci. USA 96, 435–440 (1999). 9892651 10.1073/pnas.96.2.435 PMC15154

[b39] Ohnishi H., Murata Y., Okazawa H. & Matozaki T. Src family kinases: modulators of neurotransmitter receptor function and behavior. Trends Neurosci. 34, 629–637 (2011). 22051158 10.1016/j.tins.2011.09.005

[b40] Prybylowski K. . The synaptic localization of NR2B-containing NMDA receptors is controlled by interactions with PDZ proteins and AP-2. Neuron 47, 845–857 (2005). 16157279 10.1016/j.neuron.2005.08.016 PMC1350965

[b41] Nguyen T. H., Liu J. & Lombroso P. J. Striatal enriched phosphatase 61 dephosphorylates Fyn at phosphotyrosine 420. J. Biol. Chem. 277, 24274–24279 (2002). 11983687 10.1074/jbc.M111683200

[b42] Paul S., Nairn A. C., Wang P. & Lombroso P. J. NMDA-mediated activation of the tyrosine phosphatase STEP regulates the duration of ERK signaling. Nat. Neurosci. 6, 34–42 (2003). 12483215 10.1038/nn989

[b43] Yu H. . Structural basis for the binding of proline-rich peptides to SH3 domains. Cell 76, 933–945 (1994). 7510218 10.1016/0092-8674(94)90367-0

[b44] Zarrinpar A., Bhattacharyya R. P. & Lim W. A. The structure and function of proline recognition domains. Sci. STKE 2003, RE8 (2003). 12709533 10.1126/stke.2003.179.re8

[b45] Lim W. A., Richards F. M. & Fox R. O. Structural determinants of peptide-binding orientation and of sequence specificity in SH3 domains. Nature 372, 375–379 (1994). 7802869 10.1038/372375a0

[b46] Blom N., Gammeltoft S. & Brunak S. Sequence and structure-based prediction of eukaryotic protein phosphorylation sites. J. Mol. Biol. 294, 1351–1362 (1999). 10600390 10.1006/jmbi.1999.3310

[b47] Wolf-Yadlin A., Hautaniemi S., Lauffenburger D. A. & White F. M. Multiple reaction monitoring for robust quantitative proteomic analysis of cellular signaling networks. Proc. Natl Acad. Sci. USA 104, 5860–5865 (2007). 17389395 10.1073/pnas.0608638104 PMC1851582

[b48] Collawn J. F. . Transferrin receptor internalization sequence YXRF implicates a tight turn as the structural recognition motif for endocytosis. Cell 63, 1061–1072 (1990). 2257624 10.1016/0092-8674(90)90509-d

[b49] Ohno H. . Interaction of tyrosine-based sorting signals with clathrin-associated proteins. Science 269, 1872–1875 (1995). 7569928 10.1126/science.7569928

[b50] Traub L. M. & Bonifacino J. S. Cargo recognition in clathrin-mediated endocytosis. Cold Spring Harb. Perspect. Biol. 5, a016790 (2013). 24186068 10.1101/cshperspect.a016790 PMC3809577

[b51] Boll W. . Sequence requirements for the recognition of tyrosine-based endocytic signals by clathrin AP-2 complexes. EMBO J. 15, 5789–5795 (1996). 8918456 PMC452326

[b52] Shiratori T. . Tyrosine phosphorylation controls internalization of CTLA-4 by regulating its interaction with clathrin-associated adaptor complex AP-2. Immunity 6, 583–589 (1997). 9175836 10.1016/s1074-7613(00)80346-5

[b53] Dejanovic B. . Palmitoylation of gephyrin controls receptor clustering and plasticity of GABAergic synapses. PLoS Biol. 12, e1001908 (2014). 25025157 10.1371/journal.pbio.1001908 PMC4099074

[b54] Grant S. G. . Impaired long-term potentiation, spatial learning, and hippocampal development in fyn mutant mice. Science 258, 1903–1910 (1992). 1361685 10.1126/science.1361685

[b55] Isosaka T. . Activation of Fyn tyrosine kinase in the mouse dorsal hippocampus is essential for contextual fear conditioning. Eur. J. Neurosci. 28, 973–981 (2008). 18691323 10.1111/j.1460-9568.2008.06405.x

[b56] Zhang Y. . Capping of the N-terminus of PSD-95 by calmodulin triggers its postsynaptic release. EMBO J. 33, 1341–1353 (2014). 24705785 10.1002/embj.201488126 PMC4194123

[b57] Steiner P. . Destabilization of the postsynaptic density by PSD-95 serine 73 phosphorylation inhibits spine growth and synaptic plasticity. Neuron 60, 788–802 (2008). 19081375 10.1016/j.neuron.2008.10.014 PMC2671083

[b58] Sanz-Clemente A., Matta J. A., Isaac J. T. & Roche K. W. Casein kinase 2 regulates the NR2 subunit composition of synaptic NMDA receptors. Neuron 67, 984–996 (2010). 20869595 10.1016/j.neuron.2010.08.011 PMC2947143

[b59] Setou M. . Glutamate-receptor-interacting protein GRIP1 directly steers kinesin to dendrites. Nature 417, 83–87 (2002). 11986669 10.1038/nature743

[b60] Ehlers M. D. Reinsertion or degradation of AMPA receptors determined by activity-dependent endocytic sorting. Neuron 28, 511–525 (2000). 11144360 10.1016/s0896-6273(00)00129-x

[b61] Mukai J. . Evidence that the gene encoding ZDHHC8 contributes to the risk of schizophrenia. Nat. Genet. 36, 725–731 (2004). 15184899 10.1038/ng1375

[b62] Mukai J. . Palmitoylation-dependent neurodevelopmental deficits in a mouse model of 22q11 microdeletion. Nat. Neurosci. 11, 1302–1310 (2008). 18836441 10.1038/nn.2204 PMC2756760

[b63] Stark K. L. . Altered brain microRNA biogenesis contributes to phenotypic deficits in a 22q11-deletion mouse model. Nat. Genet. 40, 751–760 (2008). 18469815 10.1038/ng.138

[b64] Israely I. . Deletion of the neuron-specific protein delta-catenin leads to severe cognitive and synaptic dysfunction. Curr. Biol. 14, 1657–1663 (2004). 15380068 10.1016/j.cub.2004.08.065

[b65] Matter C., Pribadi M., Liu X. & Trachtenberg J. T. Delta-catenin is required for the maintenance of neural structure and function in mature cortex *in vivo*. Neuron 64, 320–327 (2009). 19914181 10.1016/j.neuron.2009.09.026 PMC2840037

[b66] Xie C., Markesbery W. R. & Lovell M. A. Survival of hippocampal and cortical neurons in a mixture of MEM+ and B27-supplemented neurobasal medium. Free Radic. Biol. Med. 28, 665–672 (2000). 10754261 10.1016/s0891-5849(99)00268-3

[b67] Diering G. H., Mills F., Bamji S. X. & Numata M. Regulation of dendritic spine growth through activity-dependent recruitment of the brain-enriched Na(+)/H(+) exchanger NHE5. Mol. Biol. Cell 22, 2246–2257 (2011). 21551074 10.1091/mbc.E11-01-0066 PMC3128527

