# Supplementary material for: Activity-regulated trafficking of the palmitoyl-acyl transferase DHHC5
Source: Nat Commun. 2015 Sep 3;6:8200. doi: 10.1038/ncomms9200 (PMC4569850; doi:10.1038/ncomms9200)
Supplement: Supplementary Information — Supplementary Figures 1-5 [file ncomms9200-s1.pdf]

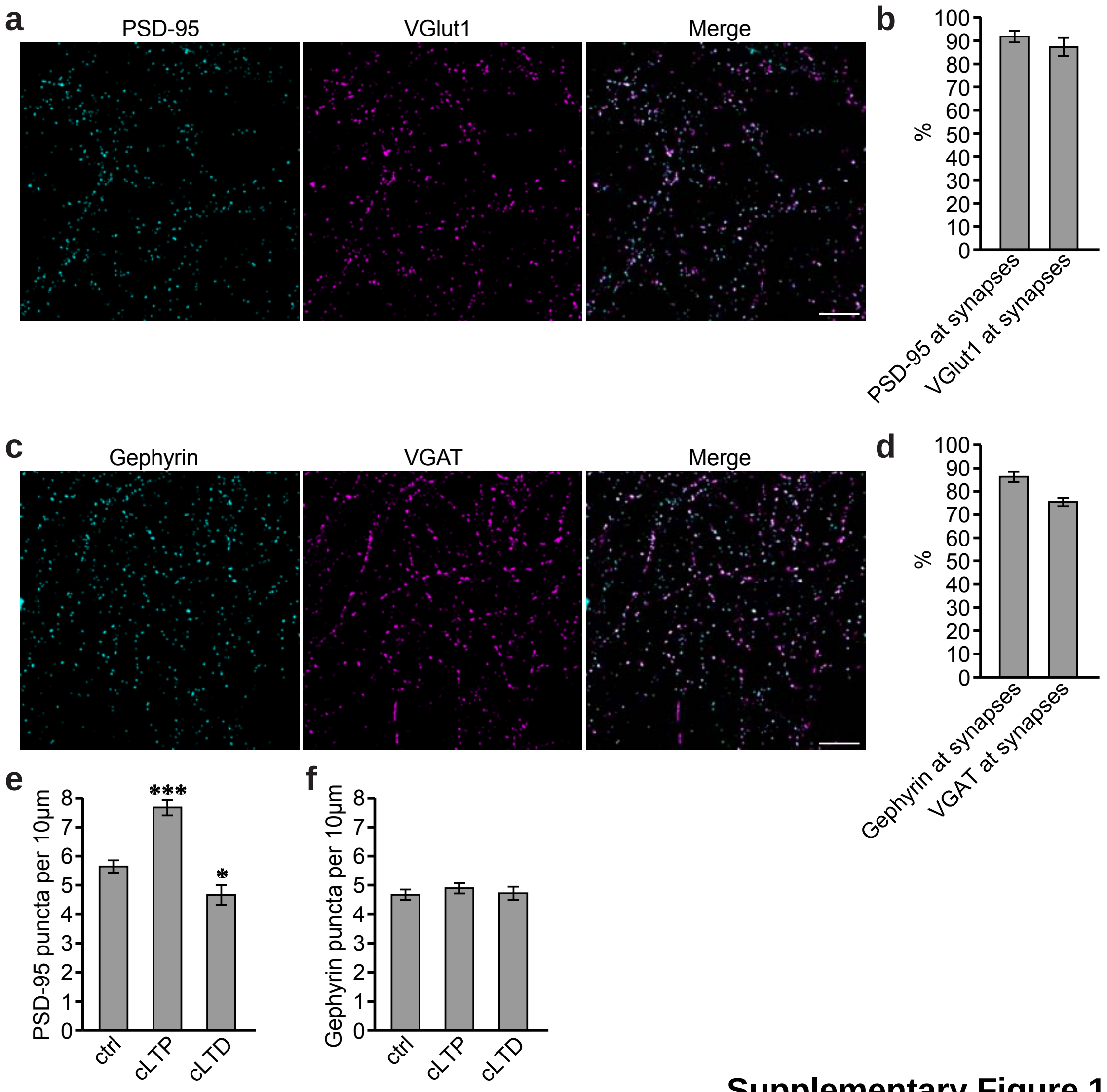

Supplementary Figure 1

Supplementary Figure 1. Pre- and postsynaptic markers of excitatory and inhibitory synapses in hippocampal neurons (**a**,**c**) Confocal images of 14 DIV neurons fixed and immunostained with antibodies against (**a**) PSD-95 and VGlut1 (merged images demarcate excitatory synapses), or (**c**) Gephyrin and VGAT (merged images demarcate inhibitory synapses). Scale bar = 20μm. (**b**) The percentage of PSD-95 at excitatory synapses was quantified by the number of colocalized PSD-95/VGlut1 puncta divided by the total number of PSD-95 puncta (91.84 ± 2.48%) and the percent VGlut1 at

excitatory synapses quantified by colocalized PSD-95/VGlut1 puncta divided by the total VGlut1 puncta (87.34 ± 3.86%; n= 10 cells). (**d**) The percentage of Gephyrin (86.34 ± 2.22%) or VGAT (75.47 ± 1.82%; n= 10 cells) at inhibitory synapses was quantified in the same manner as **b**. The density of PSD-95 puncta is altered 40 min after treatment with cLTP and cLTD relative to control cells (ctrl), ($p<0.001$, $F_{2,84}=25.82$, n=38, 35, 14), whereas the density of gephyrin is not ($p=0.665$, $F_{2,93}=0.41$, n= 37, 39, 20). *n* = cells from 3 separate cultures. All graphs display mean ± SEM. *****p<0.05, *******p<0.001; one-way ANOVA; Tukey's test *post hoc*.

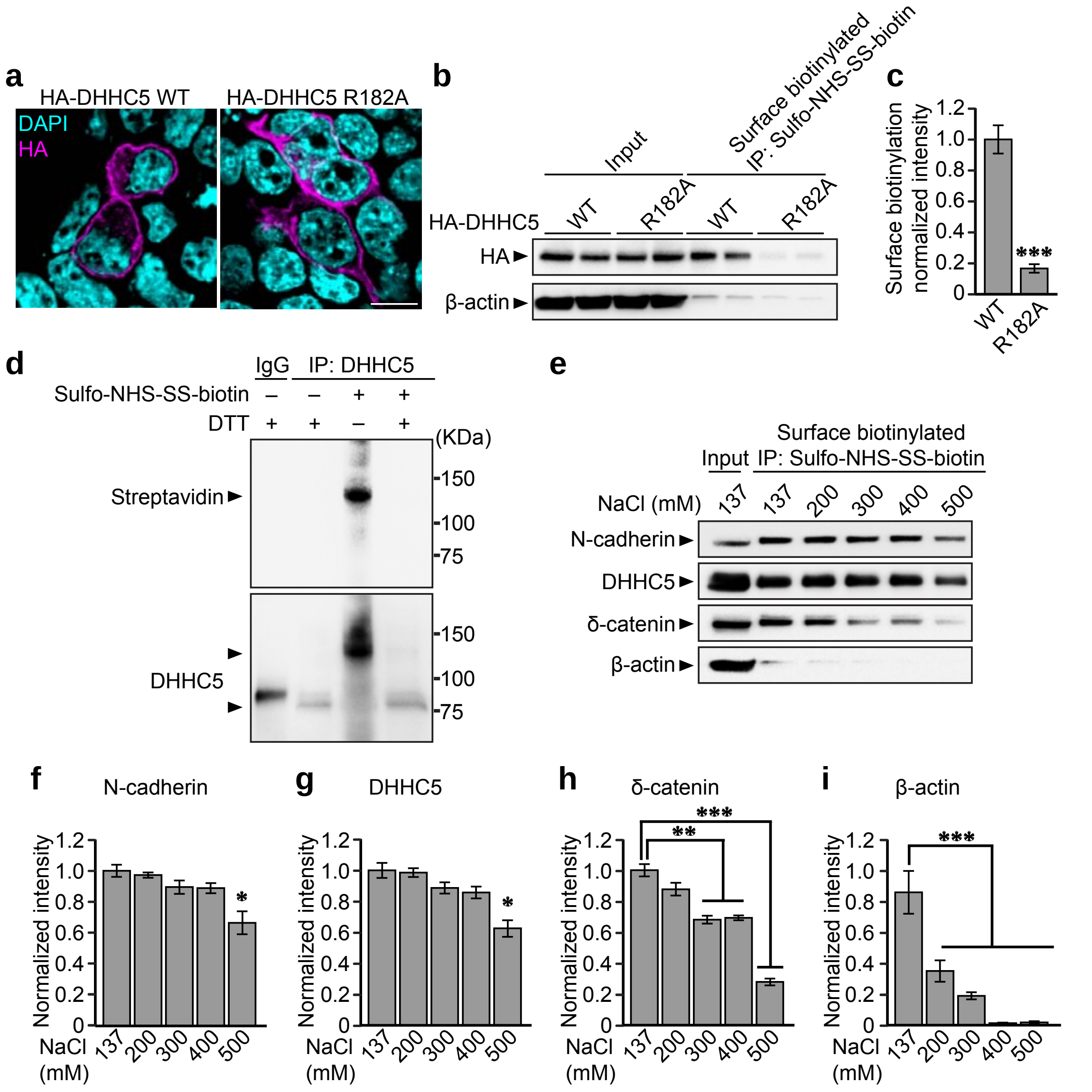

Supplementary Figure 2

Supplementary Figure 2. Surface DHHC5 is directly biotinylated at extracellular arginine R182 in hippocampal neurons. (**a-c**) HEK293T cells were transfected with HA-DHHC5 WT or R182A for 36 hrs. (**a**) Cells were fixed and immunostained with the indicated antibodies demonstrating that both are localized at the cell membrane (n = 3 separate cultures). Scale bar = 10μm. (**b**,**c**) Cells were treated with sulfo-NHS-SS-biotin, lysed, and immunoprecipitated with neutravidin-coated beads to isolate all surface

proteins. Blots were probed with the indicated antibodies. (**c**) Biotinylation of DHHC5 R182A is virtually absent compared to DHHC5 WT ($p<0.001$, student's t-test; n=3 blots from 3 separate cultures). (**d**) 14 DIV hippocampal neurons were treated with sulfo-NHS-SS-biotin or control buffer lacking biotin, lysed, immunoprecipitated with a DHHC5 antibody, and proteins were eluted in SDS-PAGE sample buffer with or without DL-Dithiothreitol (DTT; 100mM). Blots were probed with streptavidin-HRP and DHHC5 antibodies. The presence of a strepdavidin band in the –DTT, but not +DTT condition, indicates that DHHC5 is biotinylated directly. Indeed, the biotin label on immunoprecipitated DHHC5 is removed by DTT, which cleaves the disulfide bond in the sulfo-NHS-SS-biotin linker arm (n = 3 separate cultures). DHHC5 eluted in the absence of reducing agents exhibits reduced gel mobility owing to biotinylation and possibly dimerization. (**e-i**) 14 DIV neurons were surface biotinylated, lysed, immobilized on neutravidin-coated beads, and washed in standard lysis buffer containing 137mM NaCl, or increasingly stringent lysis buffers supplemented with NaCl at the indicated concentrations, prior to elution. Blots were probed with the indicated antibodies. (**f-i**) Normalized intensity of surface biotinylated. Both (**f**) N-cadherin ($p=0.0029$, $F_{4,10}=8.513$) and (**g**) DHHC5 ($p=0.005$, $F_{4,10}=13.70$) are resistant to removal from the beads by increasing NaCl titrations, owing to their direct amide bond with sulfo-NHS-SS-biotin. In contrast, (**h**) δ-catenin ($p<0.001$, $F_{4,10}=111.1$) is depleted from the IP fraction with lower NaCl concentrations, owing to its co-immunoprecipitation with surface biotinylated proteins. (**i**) The bulk cytosolic protein β-actin ($p<0.001$, $F_{4,10}=24.93$) was nearly completely absent in low salt conditions, illustrating the fidelity of the assay. 50% of whole cell lysates were loaded as inputs (n= 3 blots from 3 separate cultures). All graphs

display mean ± SEM. (**c**) *****p<0.001; student's t-test. (**f**-**i**) ****p<0.01, *****p<0.001; one-way ANOVA; Tukey's test *post hoc*.

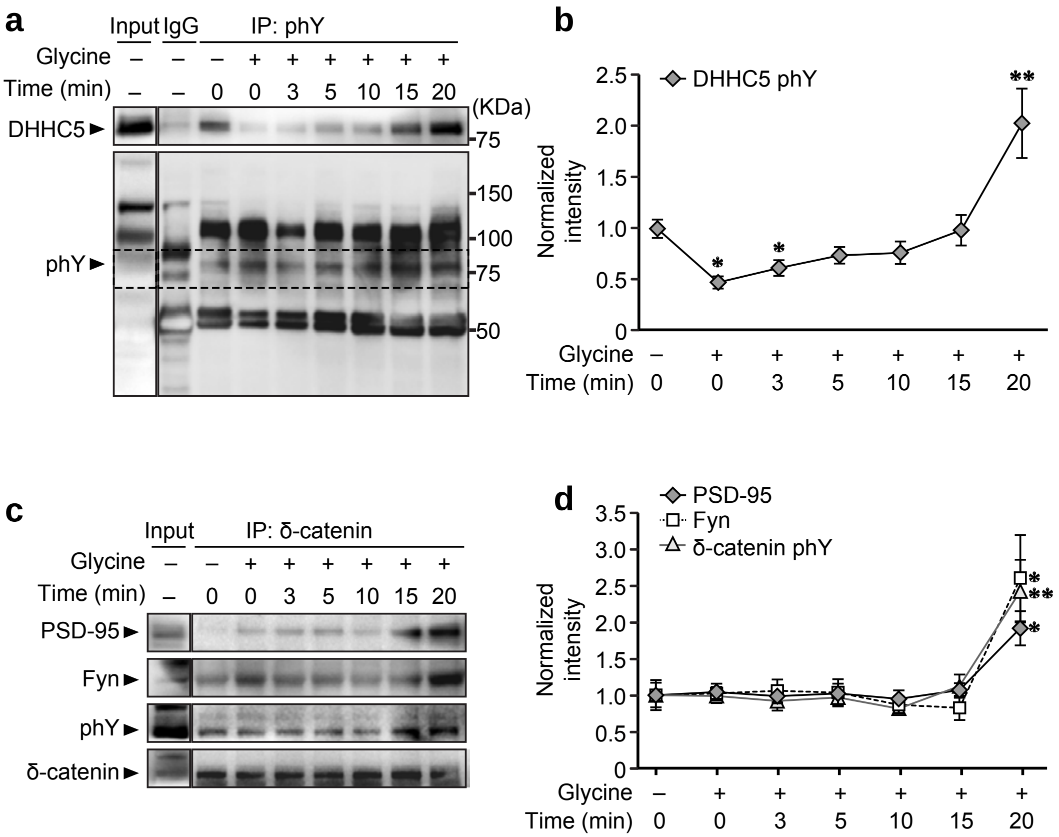

Supplementary Figure 3. Activity-regulated changes in the tyrosine phosphorylation of DHHC5 and δ-catenin. (**a-d**) 14 DIV hippocampal neurons were stimulated, lysed at the indicated time points, and lysates immunoprecipitated and blots probed with the indicated antibodies. (**a**,**b**) Tyrosine phosphorylation (phY) of DHHC5 is increased following cLTP ($p<0.001$, $F_{6,14}=11.58$; n = 3 blots from 3 separate cultures). (**c**,**d**) There is an increase in PSD-95/δ-catenin association ($p=0.013$, $F_{6,14}=7.1$), Fyn /δ-catenin association ($p=0.019$, $F_{6,14}=6.517$), and δ-catenin phY ($p=0.001$, $F_{6,14}=10.73$) following cLTP. *n* = 3 blots from 3 separate cultures. 5% of whole cell lysates were loaded as inputs, and are from the same blots but with different exposure times. Asterisks above or

next to data points indicate significance relative to unstimulated controls. All graphs display mean ± SEM. *****$p<0.05$, ******$p<0.01$; one-way ANOVA; Tukey's test *post hoc*.

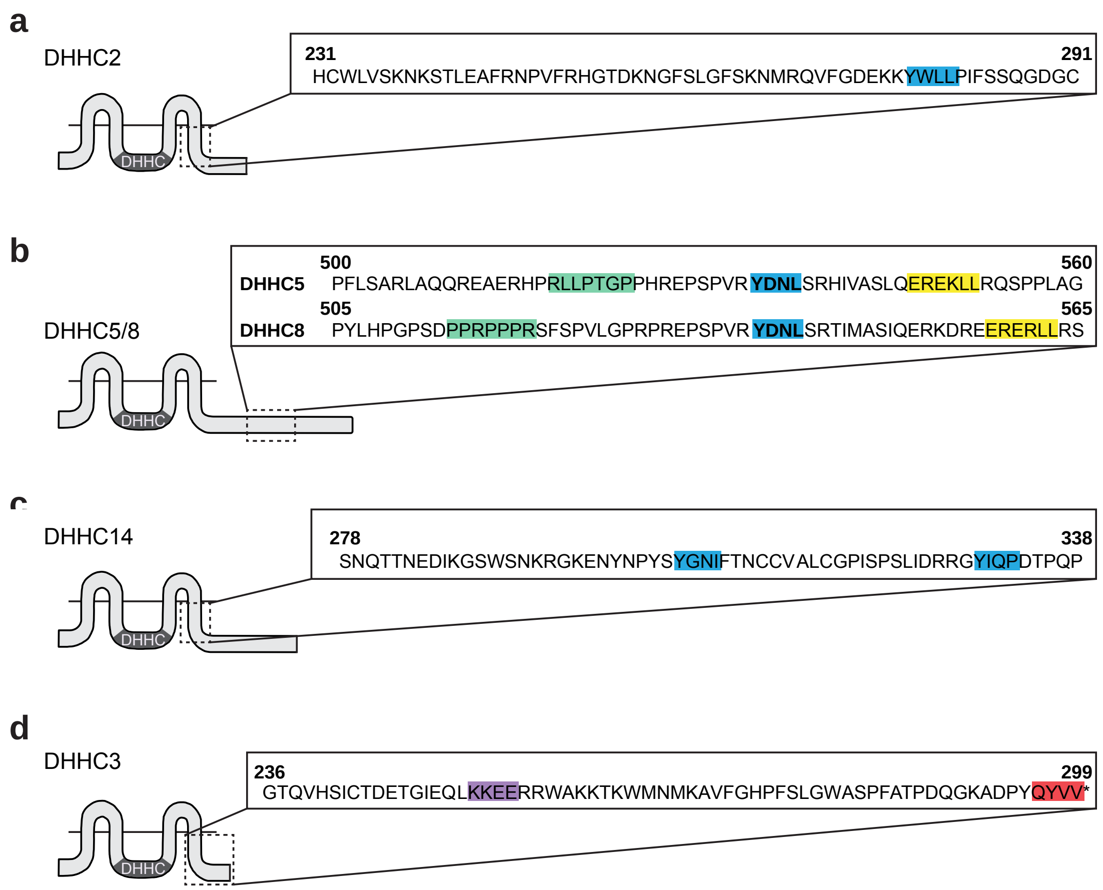

Supplementary Figure 4

Supplementary Figure 4. Presence of tyrosine-based endocytosis signals in the C-terminal tails of DHHC proteins localized to the plasma membrane. (**a-d**) C-terminal amino acid sequences of rat DHHC proteins. The dashed boxes in the schematics on the left indicate the approximate region of each protein's C-tail encompassing the amino acid sequence (numbers shown are relative to the N-terminus start for each protein). (**a**) Sequence of the membrane-proximal portion of the C-terminus of rat DHHC2 (NP_659564.2), containing a tyrosine-based endocytosis signal (highlighted in blue). (**b**) Sequence of rat DHHC5 (NP_001034427.1) aligned with that of rat DHHC8 (NP_ 001034110.1). The endocytosis motif YDNL (bold) is conserved between DHHC5 and DHHC8, and flanked by poly-proline SH3 recognition sequences (highlighted in green),

and di-leucine-based endocytosis signals (highlighted in yellow). (**c**) Sequence of the membrane-proximal portion of the C-terminus of plasma membrane-localized rat DHHC14 (NP_001034432.1), containing two tyrosine-based endocytosis signals. (**d**) Sequence of the C-terminus of DHHC3, which is localized exclusively to the somatic Golgi (NP_001034103.1). This contains a putative endoplasmic reticulum localization signal (highlighted in purple), and a PDZ-binding motif at its C-terminus (highlighted in red; asterisk indicates sequence termination). Notably, endocytic motifs are absent.

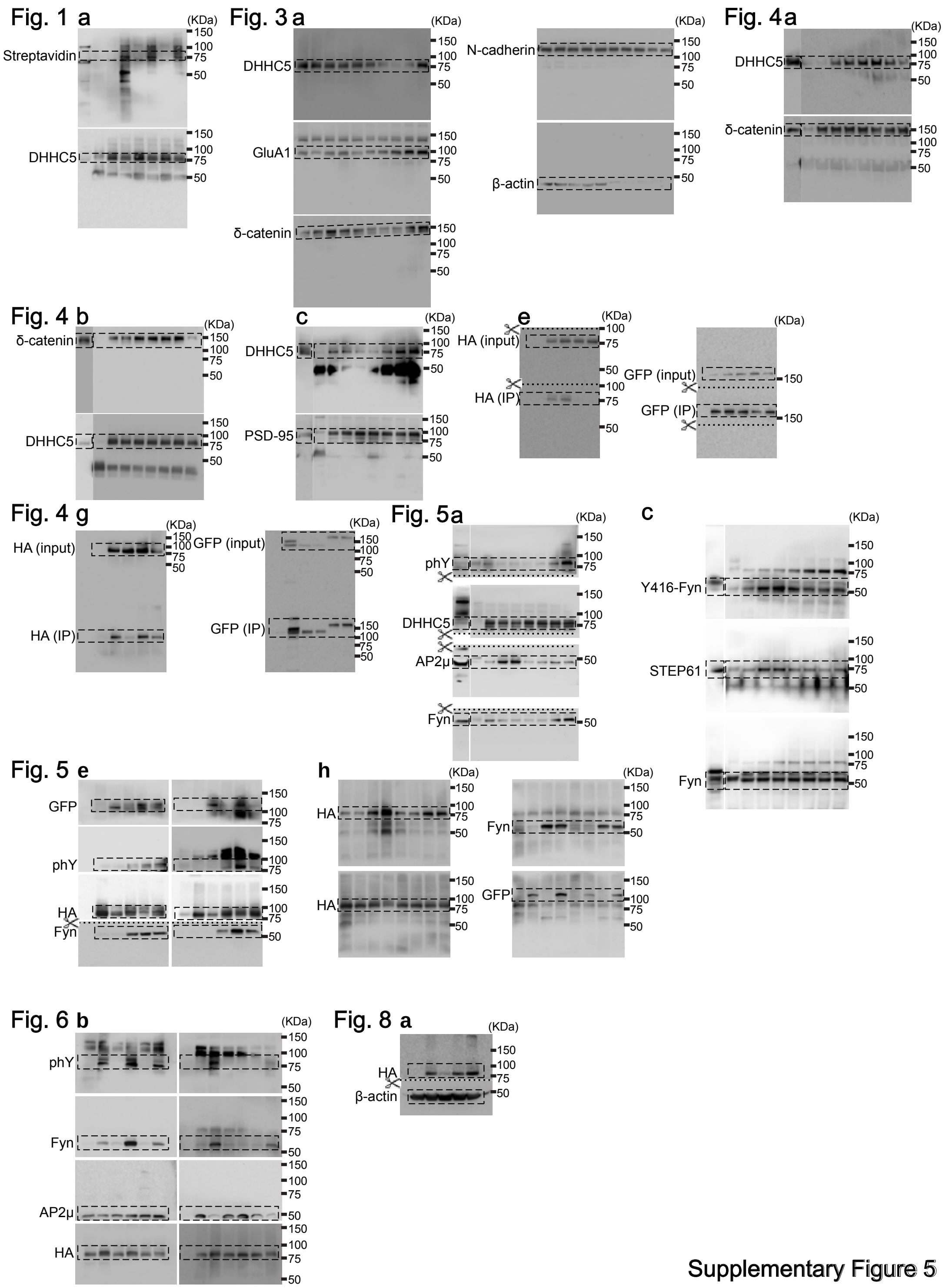

Supplementary Figure 5. Full length blots of those cropped and presented in the main figures. Scissors indicate where selected blots were cut.
